# A conserved strategy to attack collagen: The activator domain in bacterial collagenases unwinds triple-helical collagen

**DOI:** 10.1073/pnas.2321002121

**Published:** 2024-04-09

**Authors:** Jamil Serwanja, Alexander C. Wieland, Astrid Haubenhofer, Hans Brandstetter, Esther Schönauer

**Affiliations:** ^a^Department of Biosciences and Medical Biology, Paris-Lodron University of Salzburg, Salzburg A-5020, Austria; ^b^Center for Tumor Biology and Immunology (CTBI), Paris-Lodron University of Salzburg, Salzburg A-5020, Austria

**Keywords:** collagen degradation, virulence factor, *Clostridium*, bacterial enzyme, collagenase

## Abstract

Bacterial collagenases are important virulence factors, secreted by several pathogenic *Clostridium*, *Bacillus*, *Spirochaetes*, and *Vibrio* species. Yet, the mechanism by which these enzymes cleave collagen is not well understood. Based on biochemical and mutational studies we reveal that collagenase G (ColG) from *Hathewaya histolytica* recognizes and processes collagen substrates differently depending on their nature (fibrillar vs. soluble collagen); distinct dynamic interactions between the activator and peptidase domain are required based on the substrate type. Using biochemical and circular dichroism studies, we identify the presumed noncatalytic activator domain as the single-domain triple helicase that unwinds collagen locally, transiently, and reversibly.

## Significance

Collagens form the resilient backbone of the mammalian extracellular matrix. Only few proteases are able to digest collagen because of its tight triple-helical fold and high content of prolines and hydroxyprolines. Bacterial collagenases of the metalloprotease family M9 can efficiently degrade triple-helical collagen into small peptides. Yet, their mechanism of action is not well understood. We demonstrate that the activator domain of bacterial collagenases single-handedly unwinds collagen triple helices, enabling their subsequent cleavage by the peptidase domain. Our findings reveal that bacterial collagenases employ different mechanisms to recognize and unwind collagen than human collagenolytic matrix metalloproteases (MMPs). This finding opens an avenue for the development of highly selective inhibitors targeting bacterial collagenases.

Collagens are the most abundant and long-lived proteins in mammals, constituting up to 90% of the extracellular matrix (ECM). They are predominantly found as fibrillar type I collagen (~90%) (1, 2). These supramolecular assemblies shape the ECM and are essential for tissue integrity (3, 4). The basic building block of these assemblies is soluble collagen. Soluble type I collagen is a heterotrimer of two α1(I)- and one α2(I)-chains that form a right-handed triple helix flanked by short telopeptides. This triple helix is stabilized by repetitive Gly-X-Y triplets, in which X and Y are frequently occupied by proline and hydroxyproline, respectively (5). This soluble triple-helical collagen monomer (aka tropocollagen; hereafter referred to as soluble collagen) is highly resistant to proteolysis due to i) its tight helical packing, and ii) its high content of (hydroxy)prolines, which only few proteases can accommodate in their active sites (6, 7). The denatured unfolded form of soluble collagen, generated via heat denaturation or hydrolysis, is known as gelatin. Soluble collagens (~300 nm length, ~1.5 nm diameter) can self-assemble into larger fibrillar collagen complexes that are insoluble, ranging from small microfibrils (diameter of >4 nm) via fibrils (30 to 500 nm diameter) to 0.5 to 2 µm thick fibers (in sum hereafter referred to as fibrillar collagen). These fibrillar structures are further stabilized by pyridinoline crosslinks, and glycosaminoglycan interactions (4, 8–10).

A true collagenase must be able to i) bind to fibrillar collagen, ii) unwind the triple helix to excavate the scissile bonds, iii) accommodate the imino acid-rich α-chain in its active-site cleft, and iv) cleave pre- or post-(hydroxy)proline peptide bonds. Only a small number of mammalian enzymes are capable of this task under physiological conditions and typically, they do so with narrow substrate specificities (e.g., MMP-1/2/8/13/14/18, neutrophil elastase) (11, 12). However, bacteria have also evolved collagenases, most notably *Clostridium* spp., *Bacillus* spp. (M9B subfamily) and *Vibrio* spp. (M9A subfamily) (13). These bacterial enzymes are capable of cleaving soluble and fibrillar collagen down into small peptides (14–16). However, the molecular mechanisms that enable bacterial collagenases to fully degrade collagen are not completely understood.

**Fig. 1.**
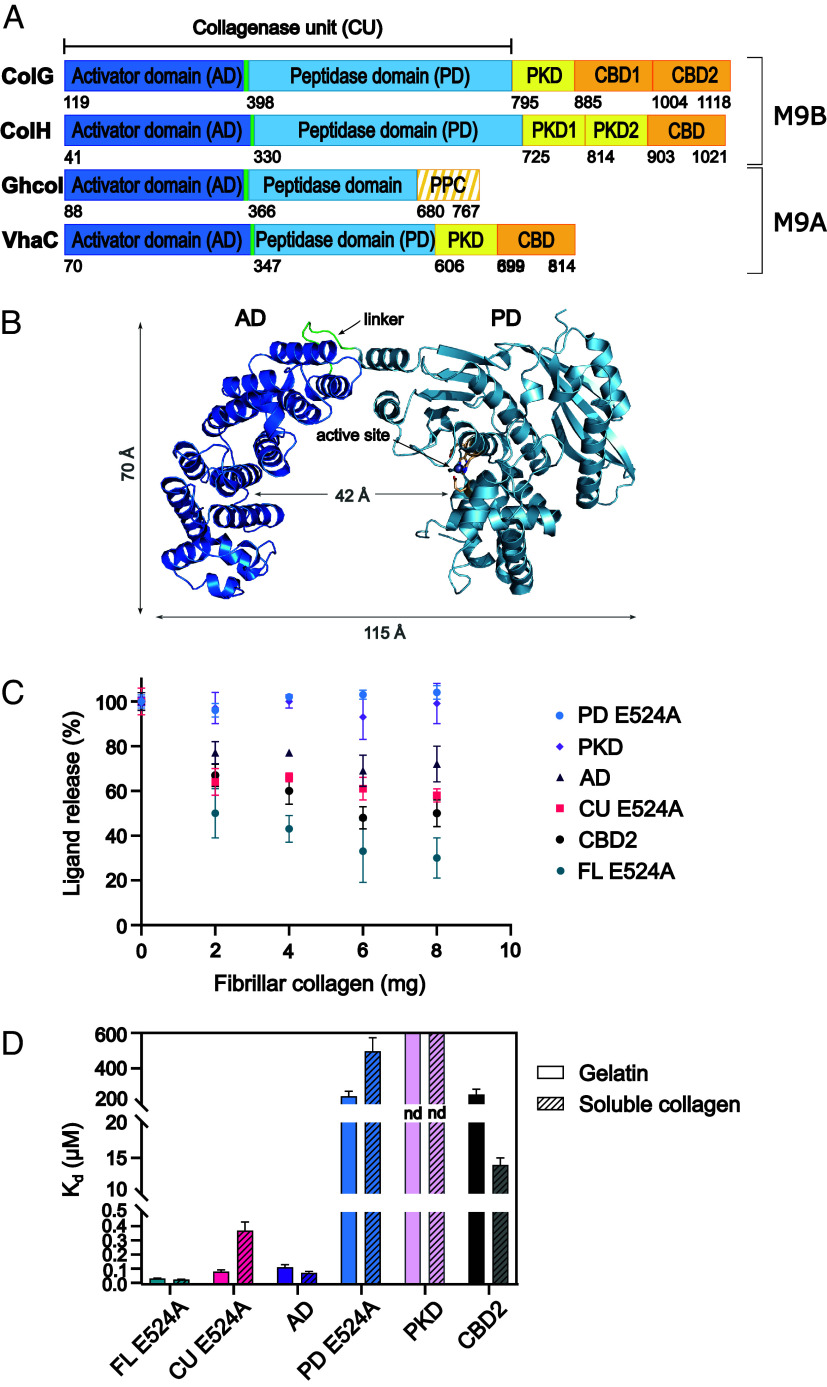
Binding of ColG variants to fibrillar collagen, soluble collagen, and gelatin. (*A*) Schematic domain organization of ColG and ColH from *H. histolytica*, Ghcol from *Grimontia hollisae*, and VhaC from *Vibrio harveyi VHJR7* (23, 27, 28). AD (dark blue), linker (light green), PD (light blue), PKD (yellow), CBD (orange), and bacterial prepeptidase C-terminal domain (orange striped) (PPC). (*B*) Ribbon representation of the CU of ColG. The catalytic zinc ion (gray) and the catalytic residues (light orange) are shown in ball-and-stick representation. The linker is highlighted (green). Molecular figures were created with PyMOL (29). (*C*) Release of fluorescently labeled ColG variants from fibrillar collagen. (*D*) Binding affinities to soluble collagen and gelatin. Apparent dissociation constants were determined via indirect ELISA, whereby the apparent K_d_ values represent the binding to gelatin or soluble collagen each with multiple binding sites per molecule. Due to very weak binding affinities, no K_d_ values could be determined for ColG-PKD. The binding curves are shown in *SI Appendix*, Fig. S1 and K_d_ values are given in *SI Appendix*, Table S1. *nd*, could not be determined.

Bacterial collagenases are multidomain zinc-metalloproteases (14, 17–21). They harbor an N-terminal collagenase unit (CU) of ~80 kDa, consisting of an activator domain (M9N domain) (AD) and a peptidase domain (peptidase M9 domain) (PD) connected via a 9 aa-long linker (17, 22, 23). At the C-terminus, a varying composition of accessory domains, i.e., polycystic-kidney disease-like domain(s) (PKD), and collagen-binding domain(s) (CBD) (each ~10 kDa), is found (17, 18, 20, 24) (Fig. 1*A*). The best studied bacterial collagenases are ColG and ColH from *Hathewaya histolytica* (formerly *Clostridium histolyticum*) (14, 17, 18, 24–26). The high-resolution structure of the CU of ColG revealed an “open” saddle-shaped CU architecture (Fig. 1*B*) and we identified the CU as the minimal collagenolytic entity in vitro (17). Based on these findings, we proposed a conformational two-state model of bacterial collagenolysis, aka chew-and-digest model, in which the processing of collagen is driven by the opening and closing of the CU. In this model, soluble collagen is recognized by the PD in the (crystallized) open CU state, thereby triggering CU closing. In the (proposed) compact state, the AD and PD are able to interact with the triple-helical collagen, thereby facilitating the unwinding and enabling the cleavage of entrapped α-chains in the (semi-) closed CU conformation (17).

Support for the existence of the closed CU conformation was provided by a SAXS model of ColH (30), and crystallographic studies identified similar open saddle-shaped CU architectures of the *Vibrio* collagenases Ghcol of *G. hollisae* (22) and VhaC of *V. harveyi VHJR7* (23). However, the authors suggested that the initial recognition of soluble collagen is mediated by the AD in bacterial collagenases (23). How bacterial collagenases manage to unfold the collagen triple helix—a feature, which sets them apart from other peptidases—and how they are able to process higher-order assemblies of collagen, these questions are still not answered.

Here, we investigate the role of individual domains of bacterial collagenases in the recognition of the different physiological forms of collagen. We identify and characterize the triple helicase in bacterial collagenases, and we demonstrate the significance of AD–PD interplay in the processing of soluble and fibrillar collagen using conformational trapping.

## Results

### Recognition of the Multihierarchical Substrate Collagen by Clostridial Collagenases.

Physiological processing of collagen involves three distinct recognition events, binding to i) insoluble fibrillar collagen; ii) soluble collagen; and iii) unfolded α-chains (i.e., gelatin). Therefore, we systematically examined the affinity of full-length ColG, its CU and of its individual domains for these three hierarchical levels of collagen. Protein variants that contained the PD carried the inactivating E524A mutation.

First, we investigated the binding of full-length ColG-FL E524A (Y119-K1118) (ColG-FL E524A) and of its domains to fibrillar collagen, the predominant physiological substrate (Fig. 1*C*). We observed concentration-dependent binding to fibers of ColG-FL E524A, ColG-CBD2 (N1004-K1118), ColG-CU E524A (Y119-G790), and ColG-AD (Y119-S392) in decreasing order, whereas very low or no binding to fibrillar collagen was seen for ColG-PD E524A (D398-G790) and ColG-PKD (I799-N880). Of all ColG-domains including the CU, ColG-CBD2 bound best to fibrillar collagen, while the PD showed no detectable binding. It is, therefore, remarkable that ColG-CU E524A showed a higher affinity to fibrillar collagen than the single AD, indicating a nonadditive, but cooperative binding mode of AD and PD to the fibrillar substrate. Together, these results suggest that the highly affine binding of the full-length enzyme is based on the high affinity of the CBD and the CU to fibrillar collagen.

Second, we examined the binding toward soluble collagen and gelatin (Fig. 1*D*). Among all variants, ColG-FL E524A displayed the highest affinity to both substrates. Intriguingly, it displayed very similar affinities toward gelatin and soluble collagen (apparent dissociation constant (K_d_) = 0.031 ± 0.004 µM vs. 0.024 ± 0.002 µM, respectively). Most of its affinity toward soluble collagen and gelatin was mediated by the CU. ColG-CU E254A bound best to gelatin (K_d_ = 0.08 ± 0.01 µM), and it showed a ~5-fold lower affinity to collagen (K_d_ = 0.37 ± 0.06 µM). Interestingly, the PD showed poor binding toward gelatin and soluble collagen, as did the accessory domains, except for CBD2. ColG-CBD2 bound to soluble collagen with a K_d_ of 14 ± 1 µM, but it displayed low binding affinity to gelatin (K_d_ = 253 ± 30 µM). In contrast, ColG-PKD exhibited very little binding to both substrates; the binding was so poor that no K_d_ values could be determined (*SI Appendix*, Fig. S1 and Table S1).

ColG-FL displayed a more than twofold higher affinity toward gelatin and a ~9-fold higher affinity to soluble collagen compared to ColG-CU. These increases in affinity result most likely from the C-terminal CBDs. In fact, the differential CBD binding affinities to soluble collagen and gelatin consistently explain the preferential collagen binding of ColG-FL, compensating for the weaker collagen binding of ColG-CU (Fig. 1*D*).

To dissect CU binding to gelatin and soluble collagen, we analyzed the binding of the individual AD and PD, which together form the CU. ColG-AD exhibited a K_d_ of 0.11 ± 0.02 µM for gelatin and an even better K_d_ of 0.072 ± 0.009 µM for soluble collagen. In contrast, ColG-PD E524A showed only low binding to gelatin (K_d_ of 243 ± 28 µM) and a twofold lower binding toward soluble collagen (K_d_ of 497 ± 76 µM). When comparing the affinities to gelatin and soluble collagen of the AD and the PD with the CU, first, it is striking that the CU binds to gelatin like the AD (K_d_ = 0.08 ± 0.01 µM vs. 0.11 ± 0.02 µM). This suggests that binding to gelatin is predominantly mediated via the AD, consistent with the several hundred-fold difference in binding affinities of AD and PD. Second and counterintuitively, CU binding to soluble collagen (K_d_ = 0.37 ± 0.06 µM) can only be accounted for an antagonistic interaction of the AD and the PD, when binding to the triple-helical substrate.

### Bacterial Collagenases Unwind Soluble Collagen Only Locally, Not Globally.

**Fig. 2.**
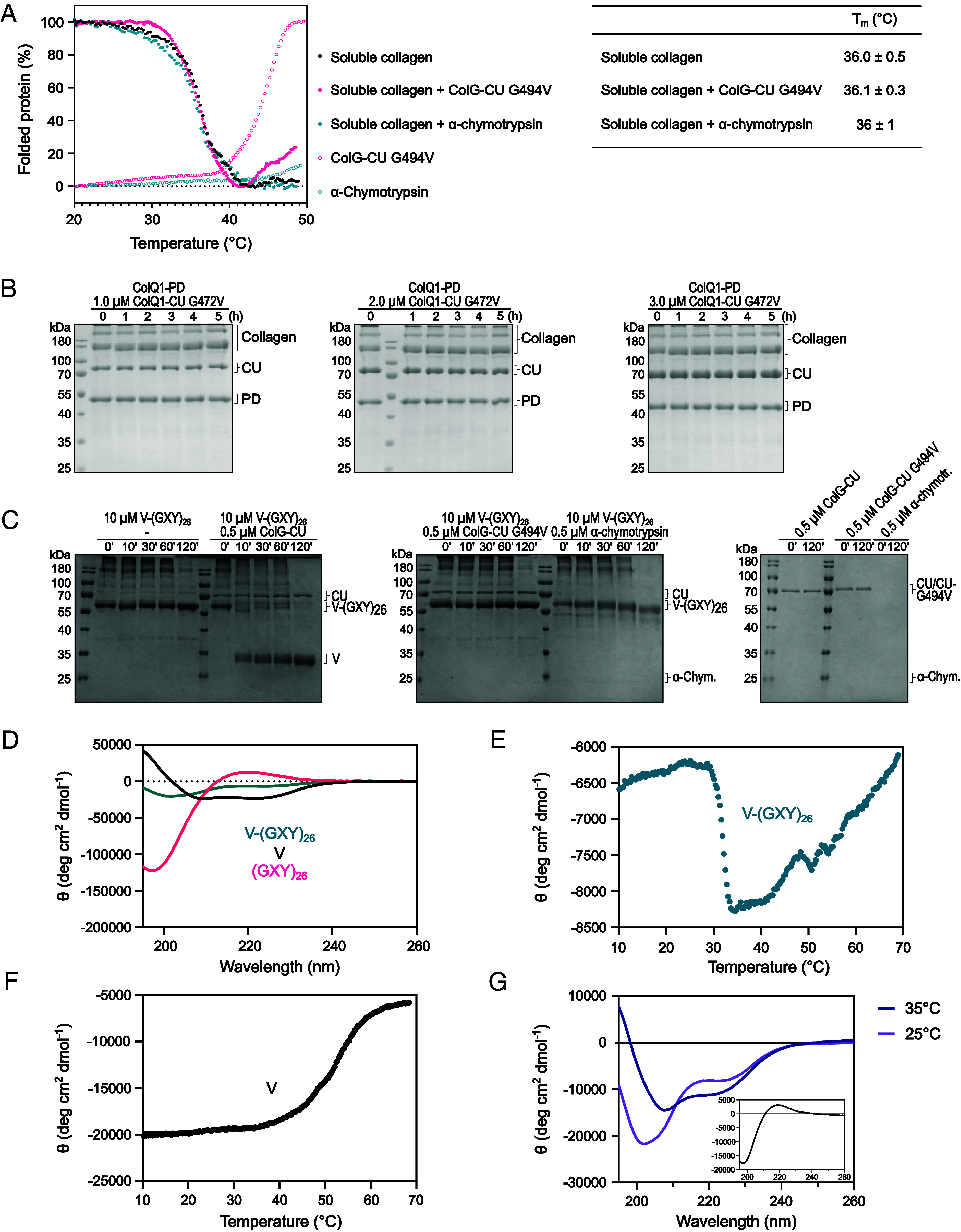
ColG does not unwind soluble collagen globally. (*A*) Thermal transition curves and melting temperatures from CD experiments for soluble collagen with or without ColG-CU G494V and α-chymotrypsin. (*B*) SDS-PAGE analysis of a degradation complementation assay, in which soluble collagen was incubated with equimolar concentrations of ColQ1-PD and increasing concentrations of ColQ1-CU G472V at 25 °C. The integrity of the substrate was verified by coincubation with 0.83 μM α-chymotrypsin. (*C*–*F*) V-(GXY)_26_ is a short collagen-like substrate. (*C*) SDS-PAGE analysis of time course of incubation of V-(GXY)_26_ with and without ColG-CU WT, ColG-CU G494V, and α-chymotrypsin. Note that the V-domain migrates as an SDS-stable trimer at an apparent mass of 30 kDa; similarly, V-(GXY)_26_ migrates as a trimer. (*D*) CD spectrum of V-(GXY)_26_ and of (GXY)_26_, prepared by pepsin digest of V_Scl2.3_-(GXY)_26_. (*E* and *F*) Melting profiles of V-(GXY)_26_ (*E*) and of the single V-domain (*F*) determined by CD spectroscopy. (*G*) CD spectra of V-(GXY)_26_ recorded at 25 and 35 °C. The inlet shows the CD difference spectrum of the 25 °C spectrum minus the 35 °C spectrum.

To investigate the triple-helicase activity of ColG, we measured the change in melting temperature (T_m_) of soluble collagen in the absence and presence of inactive ColG-CU G494V by circular dichroism (CD) spectroscopy. α-Chymotrypsin served as negative control (Fig. 2). We observed no significant change in the T_m_, neither in the absence or the presence of ColG-CU G494V, nor of α-chymotrypsin (Fig. 2*A*). This suggests that ColG does not globally unwind soluble collagen, but that the unwinding only takes place locally. Next, we performed a proteolytic complementation assay using ColQ1-PD as “cutter” enzyme and the proteolytically inactive ColQ1-CU G472V as “triple helicase” (Fig. 2*B* and *SI Appendix*, Figs. S2 and S3). ColQ1 is a bacterial collagenase from *Bacillus cereus* strain Q1 (31). Despite the addition of increasing concentrations of ColQ1-CU G472V to a constant high amount of ColQ1-PD, we observed no turnover of soluble collagen, suggesting that the unwinding of collagen by ColQ1-CU G472V only occurred locally and reversibly.

### Unwinding of Short Collagen Triple Helices Can Be Monitored via CD.

We concluded that the fraction of soluble collagen that is unwound by the collagenase is too small compared to the dimensions of the whole molecule (~300 nm in length) to have a significant effect on the overall T_m_ of soluble collagen. Therefore, we generated a short collagen-like substrate V-(GXY)_26_ to monitor the unwinding activity. This construct is derived from *Streptococcus pyogenes* collagen-like protein 2 (Scl2). It is composed of the trimerization V-domain of Scl2.3 and the proline-rich B-segment of the CL domain of Scl2.28 (32, 33). The native B-segment forms a stable collagen-like triple-helix with a T_m_ of 29.5 °C (33). We modified segment B to increase ColG’s affinity by mutating four of its tripeptides to Gly-Pro-Ala (*SI Appendix*, Fig. S4) (26). This modified construct V-(GXY)_26_ was efficiently recognized by ColG-CU. The triple-helical B-segment was readily degraded by ColG-CU, leaving the V-domain trimer intact, while the inactive G494V mutant could not cleave it. α-Chymotrypsin could only partially cleave the V-domain, but it left the triple helix intact (Fig. 2*C*).

We confirmed the triple-helical fold of the B-segment of V-(GXY)_26_ using CD (Fig. 2 *D* and *G*). The net spectrum of V-(GXY)_26_ is composed of the α-helical contribution of the V-domain (73 aa) and the triple-helical contribution of segment B (78 aa). The isolated modified B-segment, i.e., (GXY)_26_, displayed the spectral fingerprint of soluble collagen with a negative band below 200 nm and a positive band at 222 nm. When monitoring the molar ellipticity at 222 nm at temperatures between 10 and 70 °C, the melting of the V-domain (T_m_ = 52 ± 2 °C) could be clearly differentiated from the melting of the triple-helical (GXY)_26_ part (T_m_ = 31.8 ± 0.1 °C) (Fig. 2 *E* and *F*). The difference spectrum of V-(GXY)_26_ from spectra recorded at 25 and 35 °C, i.e., before and after B melting, showed CD transitions typical for the collagen triple helix (34) (Fig. 2*G*).

### The Activator Domain Is a Triple Helicase.

**Fig. 3.**
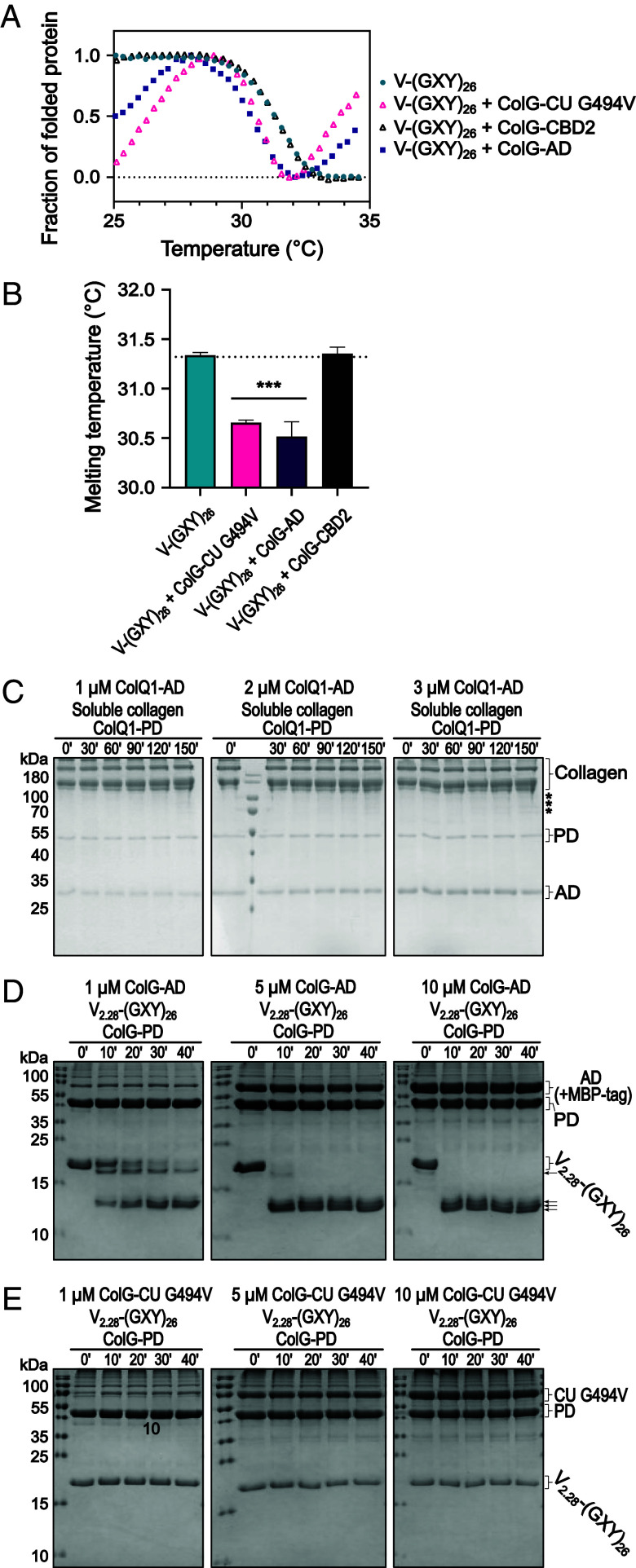
Local and transient triple-helicase activity of AD requires simultaneous colocalization of the PD for collagen cleavage. (*A*) Melting profiles of the short collagen mimic V-(GXY)_26_ in absence or presence of ColG-CU G494V, ColG-AD, and ColG-CBD2 monitored via CD spectroscopy. (*B*) Melting temperatures of V-(GXY)_26_ in absence or presence of ColG-CU G494V, ColG-AD, and ColG-CBD2 (mean ± SD). (*C*) SDS-PAGE analysis of time course of coincubation of 3.33 µM soluble collagen with increasing concentrations of ColQ1-AD (1 to 3 µM) in the presence of 0.6 µM ColQ1-PD at 25 °C. Minor fragments are highlighted with an asterisk. (*D* and *E*) SDS-PAGE analysis of time course of coincubation of 10 µM V_2.28_-(GXY)_26_, which migrates as monomer, with 1, 5 or 10 µM ColG-AD-MBP (*D*) or 1, 5 or 10 µM ColG-CU G494V (*E*) in the presence of 10 µM ColG-PD. Control reactions can be found in *SI Appendix*, Fig. S7.

To identify the triple-helicase entity in ColG, we tested all ColG domains which we had found to bind to soluble collagen (ColG-CU, ColG-AD, and ColG-CBD2) for their effect on the denaturation of V-(GXY)_26_. Remarkably, in the presence of a twofold excess of ColG-CU G494V a distinct destabilization of the triple helix was observed, causing a decrease in T_m_ from 31.3 ± 0.0 to 30.7 ± 0.0 °C (Fig. 3 *A* and *B*). As a control, we added a 6-fold excess of ColG-CBD2 to V-(GXY)_26_, which is known to bind to soluble collagen, but which cannot unwind it (17). The presence of ColG-CBD2 did not alter the T_m_ of V-(GXY)_26_ (31.4 ± 0.0 °C). With this approach, we could directly demonstrate the triple-helicase activity of bacterial collagenases.

Given that soluble collagen preferentially binds to the AD (Fig. 1*D*), we measured the melting profile of V-(GXY)_26_ in the presence of ColG-AD and found a clear decrease in T_m_ of V-(GXY)_26_ to 30.5 ± 0.2 °C, comparable to the temperature shift detected in the presence of ColG-CU G494V (Fig. 3 *A* and *B*). This finding suggests that the AD can unfold soluble collagen independently of the PD.

To confirm the triple-helicase activity of the AD, we performed two substrate degradation assays (Fig. 3 *C*–*E*). First, we monitored the cleavage of soluble collagen using ColQ1, which cleaves soluble collagen six times more efficiently than ColG (31) (Fig. 3*C* and *SI Appendix*, Fig. S5 *A*–*D*). Yet, coincubation of soluble collagen with increasing concentrations of ColQ1-AD in the presence of a constant amount of ColQ1-PD resulted only in minute degradation fragments. We hypothesized that the apparent failure of the two domains to functionally complement each other for collagen degradation was caused by the large substrate dimensions, resulting in a vast binding surface compared to the small number of enzyme molecules added.

When we repeated the assay using V_2.28_-(GXY)_26_, a modified version of V-(GXY)_26_ containing the V-domain of Scl2.28 (33), we observed a clear dose-dependent degradation of V_2.28_-(GXY)_26_, which depended on the simultaneous presence of ColG-PD as cutter and of ColG-AD as triple helicase (Fig. 3*D* and *SI Appendix*, Fig. S6 *A* and *B*). The higher local concentrations of the single AD and PD on the surface of the model collagen allowed for the productive interaction of both. These results show that the activator domain on its own was able to functionally unwind soluble collagen, i.e., the AD is the minimal entity enabling peptidolytic collagen degradation.

### AD and PD Must Simultaneously Colocalize for Efficient Catalysis of Soluble Collagen.

Inspired by complementation experiments by the Nagase group, we next tested whether a CU-dead mutant could complement an active gelatinase to degrade soluble collagen, as was successfully shown for MMP-1 (35). Therefore, we repeated the degradation assay with V_2.28_-(GXY)_26_ as substrate shown in Fig. 3*D***,** but we replaced ColG-AD as “unwinder” by ColG-CU G494V (Fig. 3*E*). Interestingly, in the presence of ColG-CU G494V the isolated ColG-PD could no longer process the collagen mimic, indicating that the unwound peptide chain(s) were not accessible to the single PD when ColG-CU G494V was bound to V_2.28_-(GXY)_26_. Consequently, the unwinding must be short-lived and reversible, as sequential unwinding by the CU-dead mutant followed by gelatinase cleavage was unproductive.

### AD Residues Critical for Soluble Collagen Binding and Unwinding.

To identify AD residues crucial for substrate recognition and unwinding, we bioinformatically analyzed M9B collagenases, followed by an alanine scan of strictly/highly conserved surface-exposed residues on the inner AD surface based on the crystal structure of ColG as the reference model (PDB: 2y6i). Seven candidate residues were identified (ColG: F148, E191, R194, Y198, Y201, N251, and F295). We generated the homologous single-point mutants of ColQ1-CU and its inactive variants ColQ1-CU G472V (ColQ1: F123, E166, R169, Y173, F176, N226, and Y270) to investigate their effects on binding to gelatin and soluble collagen, triple-helix unwinding, and hydrolysis. The structural integrity of single-point mutants was confirmed for active variants using a peptidolytic assay (*SI Appendix*, Fig. S8), and for inactive variants by nanoDSF (*SI Appendix*, Fig. S9). In addition, the proper folding of inactive variants was confirmed via CD spectra taken before melting experiments.

**Fig. 4.**
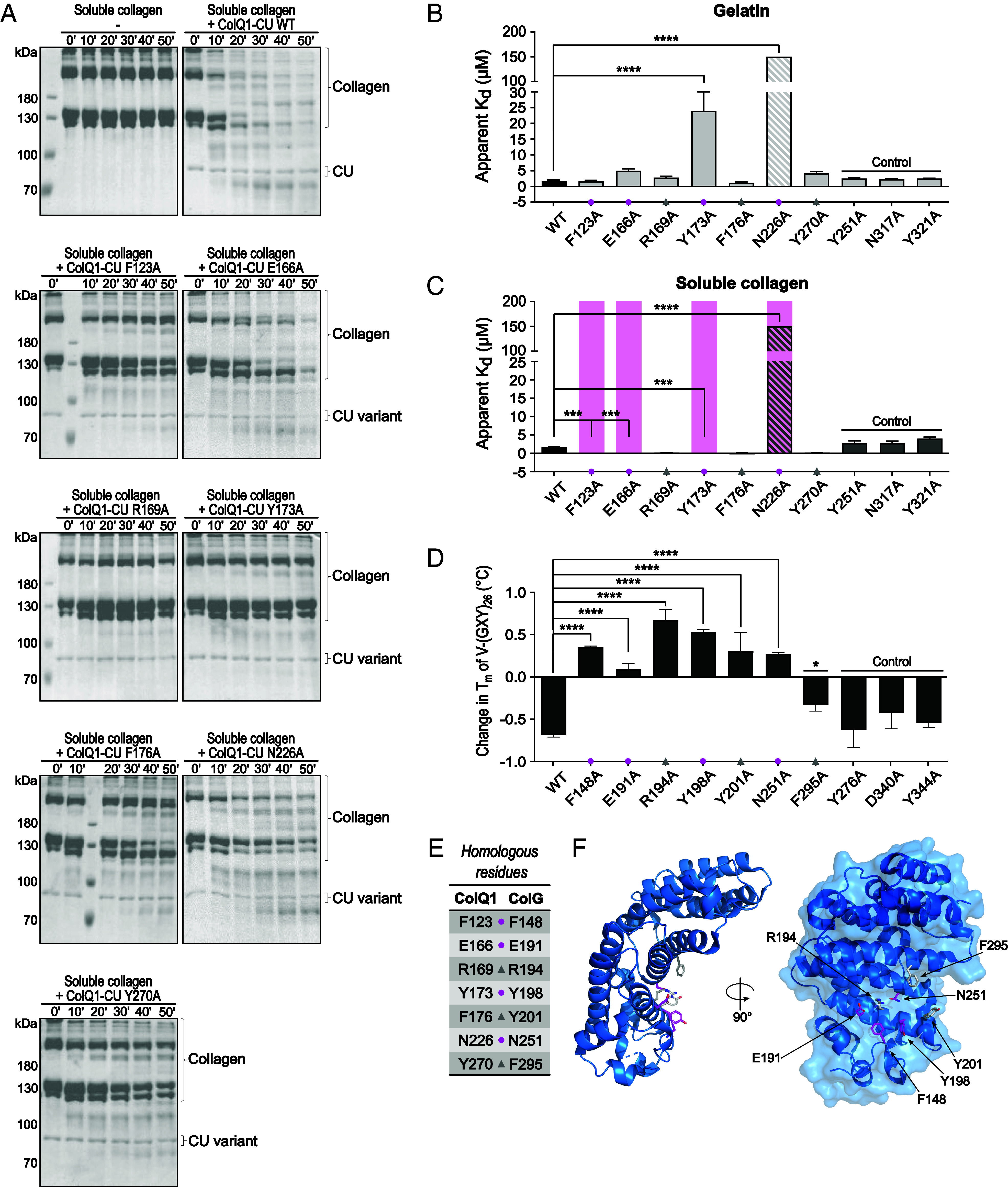
Effect of single-point mutations in AD on soluble collagen degradation, binding affinities toward gelatin and soluble collagen, and on triple-helicase activity. (*A*) Activity of ColQ1-CU WT and its mutants toward soluble collagen. SDS-PAGE analysis of time course of 3.33 µM soluble collagen coincubation with 0.2 µM ColQ1-CU variants at 25 °C. (*B* and *C*) K_d_ values of ColQ1-CU G472V (=WT) and mutants were determined by indirect ELISA. Binding affinities toward gelatin (*B*) were tested at 37 °C, while binding affinities toward soluble collagen (*C*) were tested at 25 °C to preserve the triple-helical fold. Loss of affinity was marked with a magenta bar if no binding at 100 µM concentration was detected. Mutant N226A showed no saturable binding to both substrates up to 118 µM. Therefore, its K_d_ values were estimated to be >150 µM (striped). (*D*) Change in melting temperature of V-(GXY)_26_ in the presence of ColG-CU G494V (=WT) or its mutants determined by CD spectroscopy. Homologous variants of ColQ1 and ColG are arranged on top of each other (*B*–*D*) in the figure. (*E*) Table of the homologous residues in ColQ1-AD and ColG-AD that were investigated. (*F*) Ribbon and surface representation of the AD. Residues important for soluble collagen binding, i.e., F148, E191, Y198, and N251 (magenta), and the residues crucial for unwinding, i.e., R194, Y201, and F295 (gray), are highlighted as sticks. To ease comparison between the panels, the residues involved in collagen binding (magenta circle) and unwinding (gray triangle) are marked.

Intriguingly, all seven mutants displayed impaired soluble collagen turnover in vitro, while they were able to cleave peptides like the WT (Fig. 4 and *SI Appendix*, Fig. S8). In contrast, three control mutants (ColQ1: Y251A, N317A, Y321A, also surface-exposed AD residues) did not show any aberrant collagen turnover (*SI Appendix*, Fig. S10).

To determine whether this deficiency in soluble collagen degradation was the result of binding and/or unwinding defects, we investigated i) the binding affinities toward gelatin and soluble collagen using ELISAs, and ii) the effects on the triple-helicase activity when coincubated with the CD reporter substrate V-(GXY)_26_ (Fig. 4 *B*–*F* and *SI Appendix*, Table S2).

We compared the binding of seven AD mutants to gelatin and soluble collagen to ColQ1-CU G472V (WT) (K_d_ = 1.6 ± 0.4 µM vs. 1.6 ± 0.2 µM) (Fig. 4 *B* and *C*). Two residues crucial for gelatin binding in ColQ1-AD were identified, Y173 and N226. The binding affinities to gelatin dropped drastically—15-fold and 90-fold, respectively—when these residues were replaced by alanine. Both residues were also important for soluble collagen binding. In total, four mutations resulted in significant binding defects to soluble collagen, i.e., F123A, E166A, Y173A, and N226A. Similarly to gelatin binding, the N226A mutation resulted in a more than 90-fold reduction in binding affinity to soluble collagen. Even more crucial were the mutations F123A, E166A, and Y173A, which caused a complete loss of binding. These four residues critical for soluble collagen binding and degradation cluster in the central AD cavity (Fig. 4*F*), forming a prominent exosite. In striking contrast, three other functionally inactivating mutations R169A, F176A, and Y270A resulted in a marked increase in affinity of the CU to soluble collagen of 9-fold, 20-fold, and 8-fold, respectively. To understand this, we examined the effect of AD mutations on the triple-helicase activity using the homologous mutations in inactive ColG-CU G494V (Fig. 4 *D* and *E*). The four AD mutations, which had resulted in binding defects to soluble collagen in ColQ1-CU (ColG-F148A/ColQ1-F123A, ColG-E191A/ColQ1-E166A, ColG-Y198A/ColQ1-Y173A, and ColG-N251A/ColQ1-N226A), also failed to unwind V-(GXY)_26_. This result confirms that triple-helix binding is a prerequisite for unwinding. Interestingly, ColG-R194A/ColQ1-R169A and ColG-Y201A/ColQ1-F176A, which had displayed a higher affinity to soluble collagen than the WT, were also not able to lower the T_m_ of V-(GXY)_26_ upon coincubation. This suggests that these two residues are critical for unwinding. To a lesser degree, this was also true for ColG-F295A/ColQ1-Y270A. This finding indicates that soluble collagen unwinding is not only dependent on integral binding properties, but it relies also on specific interactions which interfere directly or indirectly with the triple-helical structure, e.g., by disturbing the local hydration shell.

### Conformational Trapping of the CU Through Intramolecular Crosslinking.

**Fig. 5.**
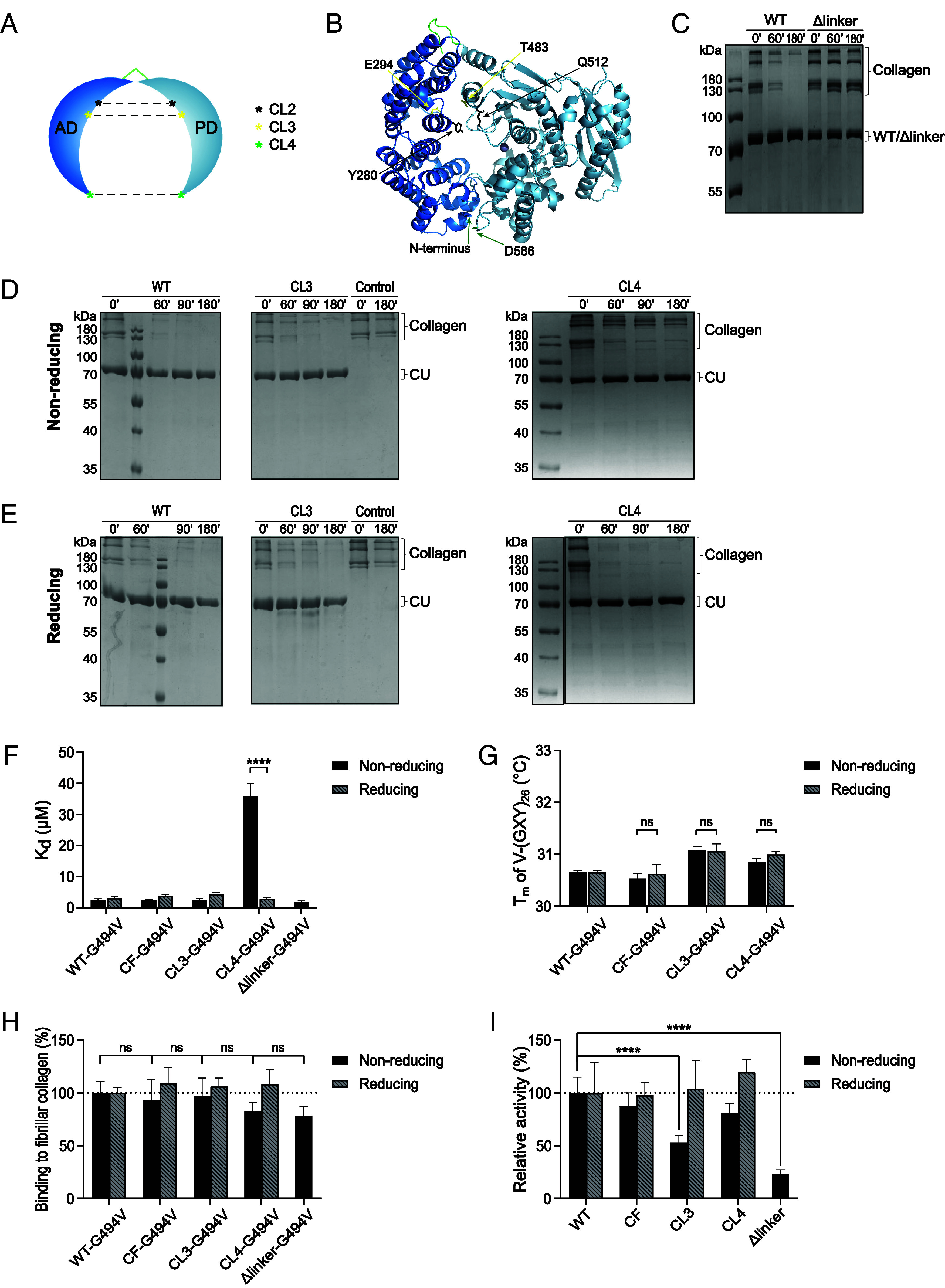
Conformational trapping and its effect on collagen degradation and binding. (*A*) Scheme of crosslinked mutants. (*B*) Model of (semi)-closed conformation of ColG-CU. Mutation sites for the introduction of cysteines are shown in sticks. (*C*–*E*) SDS-PAGE analysis of time course of degradation of soluble collagen by ColG-CU variants in absence (*C* and *D*) or presence (*E*) of 10 mM ß-mercaptoethanol. (*F*) Binding affinity of ColG-CU G494V (=WT G494V) and the mutants were determined by microscale thermophoresis. The binding curves can be found in *SI Appendix*, Fig. S15. (*G*) Change in melting temperature of the collagen mimic V-(GXY)_26_ in the presence of WT G494V and the mutants determined by CD spectroscopy. Experiments (*F* and *G*) were performed in the presence or absence of 1 mM ß-mercaptoethanol. (*H*) Binding to fibrillar collagen monitored via ligand release assay using fluorescently labeled, inactive ColG-CU. (*I*) Degradation of fibrillar collagen by crosslinked variants monitored via fluorescamine-citrate assay. Experiments (*H* and *I*) were performed in the presence or absence of 10 mM ß-mercaptoethanol.

To investigate the effect of the relative AD–PD geometry and dynamics in more detail, we generated conformationally trapped variants of ColG-CU (36–38). Conformational trapping of ColG-CU was achieved by either engineering intramolecular disulfides that crosslink the AD and the PD at various positions or by deletion of the AD–PD linker. The positions of the crosslinks were spread along the CU axis with the aim to trap the CU in a semi-closed *vs*. closed conformation (Fig. 5 *A* and *B*). We summarize the design, production, and quality assessment of crosslinked variants generated on the basis of a cysteine-free reference CF (C218S/C262S) in *SI Appendix* (39) (*SI Appendix*, Figs. S11–S14). We investigated three conformationally restrained variants, two disulfide-linked variants, ColG-CU CL3 (E294C/T483C) and CL4 (C prior to N-terminus/D586C), and a Δlinker variant (ΔG389-V397). The amidolytic activity toward short peptides was unaffected in these three conformationally restrained and the CF variants (17, 39) (*SI Appendix*, Fig. S14*D*).

### Soluble Collagen Turnover, but Not Binding, Is Significantly Reduced in the Open Conformation of the CU.

In CL3, the engineered crosslink is located ~8 Å above the level of the active-site cleft. It is designed to trap the CU in a semi-closed conformation. In CL4, where the AD and PD are linked at their tips, this will maintain a closed conformation (Fig. 5*B*); whereas the linker deletion (Δlinker) enforces a quasi-open CU conformation.

Looking at the activity of Δlinker against soluble collagen compared to the WT, we observed a drastic inhibitory effect (Fig. 5*C*). This result suggests that in the open conformation cleavage of the soluble collagen is severely hampered, most likely because substrate presentation to the PD is compromised. This conclusion is supported by the observation that the binding affinity toward soluble collagen was unchanged compared to the WT (Fig. 5*F*).

When we compared the collagenolytic activities of CL3 and CL4 in the crosslinked vs. open state, the results were remarkable (Fig. 5 *D* and *E*). The crosslink in CL3 did not compromise soluble collagen turnover, and no significant difference between crosslinked and noncrosslinked state was observed, suggesting that the semi-closed conformation did not interfere with binding and processing of soluble collagen. Only the conformationally most closed variant CL4 showed a 6-fold slower collagen turnover than the WT. This reduction in collagen turnover could be completely reversed by opening of the crosslink.

To see whether this decrease in soluble collagen cleavage resulted from impaired triple-helix binding and/or compromised helix unwinding, we examined the binding and unwinding behavior of the crosslinked mutants (CL3, CL4; CF served as control) and compared them to the reduced mutants and ColG-CU WT. We found no significant change in binding affinity to V-(GXY)_26_ in CF-G494V and CL3-G494V compared to WT-G494V, neither under nonreducing conditions, nor under reducing conditions (Fig. 5*F*). Remarkably, however, we observed a ~12-fold drop in binding affinity of CL4-G494V in the crosslinked state compared to the noncrosslinked state (K_d_ = 36 ± 4 µM vs. 2.9 ± 0.4 µM). The closed conformation led to a sharp decrease in binding affinity, explaining the observed decrease in soluble collagen turnover by CL4 in the nonreduced state.

We examined also the triple-helicase activity of the crosslinked CU variants; however, we found no ß-mercaptoethanol-dependent effect on the melting temperatures of V-(GXY)_26_ in the presence of CL3-G494V and CL4-G494V (Fig. 5*G*).

### Conformational Trapping Impairs Cleavage of Fibrillar Collagen, but Not Its Binding.

Finally, we investigated the effect of conformational trapping of ColG-CU on the recognition and processing of fibrillar collagen (Fig. 5 *H* and *I*). Importantly, we observed no significant binding defects in all mutants compared to WT-G494V, with an only marginally lower binding in crosslinked CL4-G494V and Δlinker-G494V. Thus, the closed conformation did not result in a major decrease in binding affinity. Apparently, in the disulfide-restrained CU synergistic AD–PD interactions with fibrillar collagen were able to compensate for effects of impaired cooperativity between the AD and the PD in the context of soluble collagen (Figs. 1*C* and 5*H*).

Regarding the processing of fibrillar collagen by the mutants, the findings were remarkable. Δlinker with its locked-open conformation showed a drastic reduction of 77 ± 4% in fibrillar collagen cleavage compared to the WT (Fig. 5*I*), consistent with a similarly reduced cleavage of soluble collagen (Fig. 5*C*). CL3, trapped in a semi-closed conformation, showed also a notable 47 ± 7% decrease in fibrillar collagen processing, which could be completely reversed by the addition of ß-mercaptoethanol. Finally, CL4 exhibited a 19 ± 9% reduction in activity in the crosslinked state, and full activity could be recovered upon disulfide reduction, as in CL3. All three conformationally restrained variants demonstrate the necessity of interdomain dynamics for fibrillar collagen degradation, but not for binding.

## Discussion

**Fig. 6.**
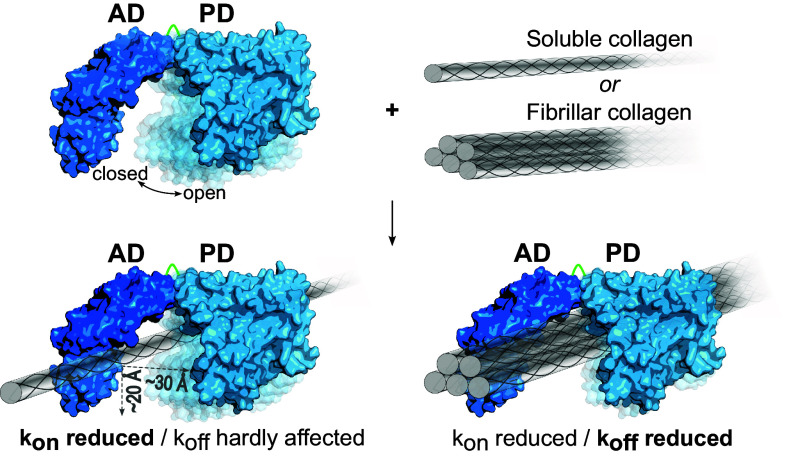
Schematic model of CU binding to soluble and fibrillar collagen illustrating antagonistic and synergistic effects. The PD sterically restricts access to AD binding sites in the saddle-shaped CU for soluble collagen molecules and fibrillar collagen (illustrated by a microfibril). Only suitably preoriented molecules can access and bind, resulting in an overall reduction of k_on_. Compared to the AD, the affinity of the CU toward soluble collagen is lowered, because the presence of the PD hardly diminishes k_off_ for the relatively small triple helix, whereas the saddle-shaped CU can “cage” the considerably larger microfibril, resulting in a substantial k_off_-effect and thus in an overall higher affinity of the CU for microfibrils.

The AD dominates the binding interaction toward soluble collagen, whereas the isolated PD shows only negligible binding affinity to fibrillar and soluble collagen. It was, therefore, unexpected to see that the binding-incompetent PD significantly impacts collagen binding in the context of the CU. Even more surprising, the effect had an opposite sign for soluble and fibrillar collagen binding. While the PD and AD interact synergistically to increase the binding affinity of the CU to fibrillar collagen (Fig. 1*C*), the opposite is true for soluble collagen (Fig. 1*D*), where the PD antagonizes AD binding. These apparently counterintuitive observations must be rationalized mostly by entropic considerations, as the PD’s enthalpic contributions to binding are negligible (Fig. 6). The binding affinity is determined by the ratio of the on-rate (k_on_) and the off-rate (k_off_). The presence of the PD abates the diffusion-controlled accessibility of large rigid macromolecular ligands such as soluble or fibrillar collagen, but not that of small or unfolded flexible macromolecular ligands such as gelatin, where the locally controlled diffusion experiences no steric restrictions to access the AD. Only suitably preoriented soluble collagen monomers can access the saddle-shaped topology of the CU and bind to the AD. This conformational selection reduces the k_on_, and it represents a significant entropic cost for the binding of soluble collagen. This effect is further enhanced when considering that AD binding to collagen is most likely a multistep process, where an initial docking geometry is subsequently adjusted for optimal interaction. The presence of the PD sterically interferes with this multistep docking process. To avoid misconceptions, we wish to make it clear that the CU will be significantly more mobile than collagen, reflecting their different hydrodynamic radii. However, since only relative movements are relevant in the docking process and it is more conventional to assume that the substrate moves and binds to the enzyme receptor, we adhere to this linguistic convention.

However, the situation differs for the off-rate in case of soluble and fibrillar collagen (Fig. 6). In case of the AD-bound soluble collagen, the k_off_ is hardly reduced by the presence of the PD. The diffusion distances to leave the CU saddle are comparable to those, where collisions with the PD could significantly hinder and slow down soluble collagen to diffuse out of the CU saddle. Taken both effects together, the affinity (i.e., the ratio k_on_/k_off_) of CU toward soluble collagen is reduced. By contrast, the saddle-shaped topology of the CU acts as a cage for bound fibrillar collagen. Most diffusion events away from the AD collide with the PD, thus slowing down k_off_ significantly, more than k_on_ is reduced by the presence of the PD. The selective effect of the CU topology on k_off_, and the overall reduction of k_on_ thus explain the observed antagonistic effect toward conformationally rigid collagen triple helices and the synergistic effect toward fibrillar collagen.

AD’s prominent role is not limited to collagen binding. We showed here that the AD can unwind collagen triple helices. Monitoring AD-induced unfolding of soluble collagen is challenging because the unfolding is locally restricted, transient, and reversible (Figs. 2 and 3), but it became feasible by developing a minicollagen reporter for CD spectroscopy. Moreover, we identified and characterized individual AD sites, which inactivated its unwinding activity either by loss of collagen binding or by gain of collagen binding and triple-helix stabilization (Fig. 4). While four residues in the AD abolished soluble collagen binding and unfolding, we identified three AD residues, which did not interfere with soluble collagen binding, but proved vital for the unwinding of soluble collagen. When mutated to alanine (ColG-R194A/ColQ1-R169A, ColG-Y201A/ColQ1-F176A, and ColG-F295A/ColQ1-Y270A), collagenolytic activity was abolished. Our structural analysis of the AD exosite, in which the seven residues crucial for soluble collagen binding and unwinding cluster, reveals deep binding pockets. This strongly suggests that the AD is a druggable domain, which is consistent with findings by Wang et al. (23) and Ikeuchi et al. (22). Inhibitory compounds targeting the exosite in the AD instead of the active site would hold the promise of minimal off-target activity. Currently, most bacterial collagenase inhibitors suffer from poor selectivity for bacterial collagenases over human MMPs, because the active-site topologies of the peptidase domains in both families are very similar (40).

Finally, we tested whether the found synergistic and antagonistic domain interactions were consistent with the proposed chew-and-digest model (17). To this end, we used conformationally restrained CU variants, which either locked the CU in an open state by minimizing the linker between AD and PD or in more closed CU conformations by disulfide linking the AD with the PD (Fig. 5 *A* and *B*). Overall, the relative flexibility of the AD–PD linkage was critical for processing of soluble and fibrillar collagen substrates, as their degradation was largely abolished by the locked-open Δlinker variant, although its binding was unaffected. Mechanistic details of collagen processing depend on the collagen conformer serving as substrate. When analyzing the disulfide-locked variants, the reduced AD–PD interdomain flexibility affected the processing of soluble collagen less severely than that of fibrillar collagen (Fig. 5). These findings can be explained by a modified chew-and-digest mechanism of bacterial collagenolysis. The Δlinker and the disulfide crosslinked variant CL4 restrain the CU in two opposing conformations, i.e., open and closed, respectively. The locked-closed conformation (crosslinked CL4) keeps the collagenase “mouth” closed, thereby restraining the chewing amplitude, but not preventing substrate chewing altogether. Low amplitude chewing allows for crunching of relatively small substrates (soluble collagen), but much less so of larger substrates (fibrillar collagen), consistent with our findings. By contrast, in the locked-open conformation (Δlinker), chewing is impossible and so is processing. Although intuitively plausible, why is collagen processing impossible in the locked-open collagenase? After all, substrate binding to the AD is unaffected, and AD binding is sufficient to induce collagen unwinding. So whenever unwound collagen dissociates from the AD, the PD should be able to cleave the prepared, gelatin-like substrate. However, this was not observed, because of the transient and reversible nature of collagen unwinding. As soon as the locally unwound collagen dissociates from the AD, it instantaneously refolds to its triple helical conformation. For productive cleavage, it is necessary to deliver the AD-bound collagen to the active site of the PD. This conclusion is consistent with the results of the complementation experiment, where an AD, but not an inactive CU, could feed unfolded collagen productively to an active PD (Fig. 3 *C*–*E*). The inactive CU locally unfolds the collagen but at the same time protects it from cleavage, because its inactive PD shields the unfolded substrate.

This finding also demonstrates that binding, unwinding, and substrate presentation of soluble collagen decisively differ between collagenolytic MMPs and bacterial collagenases, while they share some basic features. In both, the catalytic domains require the help of an additional activating domain to be able to process soluble collagen. In both families, the peptidase domains alone possess little affinity for folded collagen (41). And in both enzyme families, collagen unwinding is locally restricted (35), yet distinctly different. In MMP-1, the hemopexin (Hpx) and catalytic domains (Cat) bind in sequence to soluble collagen. When both domains are bound, a back rotation of the catalytic domain causes the release of one α-chain from the triple-helical assembly (42), illustrating that triple-helix unwinding is a two-domain job in MMPs. Moreover, both domains bind to collagen in an elongated conformation, which leaves the surface of the bound substrate largely exposed (42). Consistent with this finding, the single catalytic domain of MMP-1 added as cutter enzyme could cleave soluble collagen when preincubated and unwound by the inactivate full-length MMP-1 E200A (35). The same is, however, not true for bacterial collagenases. Here, access to the by the AD unwound α-chain(s) is blocked in the CU, because the substrate is sandwiched between the AD and the PD (*SI Appendix*, Fig. S16). This contracted conformation is required for proper presentation of the α-chains by the AD to the PD for cleavage.

## Conclusion

Within multidomain enzymes, the catalytic domain is usually considered to be the most important domain because it contains the active site, where substrate processing takes place. Our data presented here provide a much more resolved picture of substrate recognition and processing in collagenases, reflecting the complexity of collagen substrates. The AD initiates collagen binding (together with the CBDs), unfolds collagen by destabilizing its triple-helical structure, and presents the locally unwound collagen to the active site of the PD. This mechanism of collagen triple-helix binding and unwinding by a single-domain is completely distinct from the two-domain mechanism employed by mammalian MMPs. Therefore, this finding opens an avenue for the development of highly specific antivirulence agents with minimal off-target activity against bacterial collagenases secreted by pathogens. While substantial cooperation between the AD and PD was to be expected in collagen processing, it was a complete surprise to see synergistic and antagonistic cooperativity for fibrillar and soluble collagen, respectively. The here-obtained deeper mechanistic understanding of substrate selectivity paves the way for the design of collagenases with unprecedented specificities. Such uniquely specific enzymes can be used for various biotechnological applications in wound healing (43), oncolysis (44), the treatment of fibromatoses (45, 46), and cosmetic surgery (47), to name a few.

## Materials and Methods

### Materials.

Acid-soluble type I atelocollagen from bovine hides was purchased from Cell Guidance Systems (UK). Type I gelatin was produced by heating the acid-soluble type I atelocollagen for 5 min to 95 °C. Type I collagen fibers from bovine Achilles tendon were purchased from Merck (Germany).

### In Silico Analysis of the AD in Bacterial Collagenases.

Multiple-sequence alignment of the AD of ColA from *Clostridium perfringens* (P43153), ColG from *Clostridium botulinum* (B2TJU5), ColT from *Clostridium tetani* (Q899Y1), ColG and ColH from *Clostridium histolyticum* (Q9X721 & Q46085), ColQ1 from *B. cereus* (B9J3S4) were performed using Clustal Omega (48).

### Construction of V-(GXY)_26_.

The coding sequences of V-B from Scl2.28 incorporating the 4 mutated tripeptides and the coding sequence of the V domain from Scl2.3 were purchased from Genscript (Germany). The modified coding sequence of V-B was cloned into a modified pET15b vector. The endogenous V domain was replaced by the V domain from Scl2.3 via Gibson assembly. All constructs were verified by sequencing at Eurofins Genomics (Germany).

### Construction of ColG-CU Variants.

Based on a pET15b expression plasmid of ColG-CU WT (Tyr119–Gly790) (17), a plasmid encoding a cysteine-free ColG-CU variant (CF) (C218S, C262S) was generated by Gibson assembly. Using site-directed mutagenesis via inverse PCR, the plasmids for the ColG variants CL1, CL2, CL3, and CL4 were obtained from the cysteine-free template, while the linker variants were generated from the plasmid encoding ColG-CU WT. All constructs were verified by sequencing at Eurofins Genomics (Germany).

### Expression and Purification of ColG Variants, ColQ1 Variants and V-(GXY)_26_.

All protein variants were expressed in *Escherichia coli* Nico21 (DE3) cells and purified as described previously (26, 31). Monodisperse protein fractions were collected and stored at −80 °C, and the sample purity was confirmed by SDS-PAGE analysis under denaturing conditions.

### Preparation of (GXY)_26_.

11 µM V-(GXY)_26_ were coincubated in a molar ratio of 1:20 with pepsin in 50 mM acetic acid pH 3.0 at 4 °C for 72 h. The reaction was stopped by pH adjustment to pH 7.4 and the addition of 10 mM ß-mercaptoethanol. The structural integrity of (GXY)_26_ was confirmed by coincubation with α-chymotrypsin in a molar ratio 1:10 for up to 3 h.

### Disulfide Formation and Thiol Quantification Assay.

For the crosslinkage, purified collagenases (0.1 mg/mL final concentration) were suspended in 50 mM Tris-HCl pH 8.5, 1 mM β-mercaptoethanol, 300 mM NaCl, 5% glycerol, 1 mM CaCl_2_ and 3 mM NaN_3_. The oxidation reactions were kept at 4 °C for 10 d followed by centrifugation (16,500×*g* for 20 min). The nonoxidized molecules were removed by Activated thiol sepharose^TM^ 4B chromatography (Sigma Aldrich) performed according to the manufacturer’s recommendations. The crosslinked monomers were separated from misoxidized aggregates by size exclusion chromatography. The extent of disulfide-bridge formation in the samples CL1-CL4 was examined using the thiol-specific fluorochrome 7-diethylamino-3-(4-maleinimidophenyl)-4-methyl coumarin (CPM) and ColG-CU WT (contains 2 cysteines) as positive control. More details can be found in *SI Appendix*.

### CD Spectroscopy.

Far UV CD spectra in the wavelength range from 195 to 260 nm were recorded using a Chirascan Plus CD Spectrophotometer (Applied Photophysics, Leatherhead, UK), equipped with a Peltier temperature-controlled cuvette holder at 25 °C in a 0.5-mm path length quartz cuvette. The samples (2.25 µM type I collagen, 5.0 µM V-(GXY)_26_, 5.0 µM V, 2.25 µM and 10.0 µM ColG-CU G494V, 30.0 µM ColG-CBD2, 2.25 µM α-chymotrypsin, 10 µM ColG-CU G494V mutants, 10.0 µM CF, and 10.0 µM CL1–CL4) were measured in 15 mM Tris-SO_4_ pH 7.5, 100 mM NaF, 1 mM CaCl_2_ and ±1 mM ß-mercaptoethanol. Melting profiles were measured at similar concentrations at a wavelength of 222 nm. More details can be found in *SI Appendix*.

### Determination of Thermal Stability.

Thermal denaturation assays were performed using Tycho NT. 6 (NanoTemper Technologies, Germany). The measurements were performed at 0.1 mg/mL protein concentration in 15 mM Tris-SO_4_ pH 7.5, 100 mM NaF and 1 mM CaCl_2_ in triplicates. Intrinsic fluorescence was recorded at 330 and 350 nm while heating the sample from 35 to 95 °C at a rate of 30 °C/min. Fluorescence ratio (350/330 nm) and inflection temperature were calculated by Tycho NT.6 software.

### Peptidolytic Assay.

The peptide-degradation assay was performed as described previously (40). Details can be found in *SI Appendix*.

### Degradation of V-(GXY)_26_ Monitored via SDS–PAGE.

10 µM V-(GXY)_26_ were digested at 25 °C by 0.5 µM ColG-CU or 0.5 µM α-chymotrypsin in 250 mM HEPES pH 7.5, 400 mM NaCl, 10 mM CaCl_2_, and 10 µM ZnCl_2_ for up to 2 h at 25 °C. Samples were taken at indicated time points and the reaction was stopped by addition of SDS–PAGE loading buffer on ice. The degradation was monitored using 12% nonreducing SDS–PAGE gels.

### Degradation of V_2.28_-(GXY)_26_ Monitored via SDS–PAGE.

10 µM V_2.28_-(GXY)_26_ were coincubated with 1, 5 or 10 µM ColG-AD-MBP in the presence or absence of 10 µM ColG-PD in 250 mM HEPES pH 7.5, 400 mM NaCl, 10 mM CaCl_2_, and 10 µM ZnCl_2_ for up to 40 min at 25 °C. Samples were taken at indicated time points and the reaction was stopped by addition of SDS–PAGE loading buffer on ice. The degradation was monitored using 16% nonreducing SDS–PAGE gels.

### Degradation of Soluble Collagen Monitored via SDS–PAGE.

3.33 µM acid-soluble type I atelocollagen from bovine hides (Cell Guidance Systems, Cambridge, UK) was digested at 25 °C by 4.54 µM collagenase in 250 mM HEPES pH 7.5, 400 mM NaCl, 10 mM CaCl_2_, 10 µM ZnCl_2_, and ±10 mM ß-mercaptoethanol for up to 4 h. Samples were taken at indicated time points and the reaction was stopped by addition of 38 mM EDTA. The integrity of the triple-helical collagen fold was verified by coincubation with 0.83 µM α-chymotrypsin (FLUKA, Switzerland). The degradation was monitored on 12% nonreducing SDS–PAGE gels. All experiments were performed at least in triplicates. Densitometric analysis was performed using GelAnalyzer 19.1 (www.gelanalyzer.com).

### Degradation of Fibrillar Collagen Monitored via Fluorescamine-Citrate Assay.

Two milligrams of fibrillar collagen from bovine Achilles tendon (Merck, Germany) were added to a Nanosep microcentrifugal device (0.2 µm pore size) with a low-binding Bio-Inert membrane (Pall, Germany). Subsequently, 500 µL reaction buffer (250 mM HEPES pH 7.5, 400 mM NaCl, 10 mM CaCl_2_, and 10 µM ZnCl_2_, ±10 mM ß-mercaptoethanol) were added for 15 min at room temperature to swell the fibers and then removed via centrifugation (13,000×*g* for 2 min). Then, 200 µL 1.0 µM ColG-CU variants were added and incubated for 2 h at room temperature. The filtrate was collected and supplemented with 38 mM EDTA to stop the reaction. The amount of hydrolysis was quantified in comparison to the control reaction with ColG-CU WT exploiting the N-terminal-specific adduction of fluorescamine to peptides proteins at mildly acidic pH (49). All experiments were performed at least in triplicates. More details can be found in *SI Appendix*.

### Indirect ELISA.

Since gelatin can partially refold into triple-helical structures upon cooling (50), particular care was taken to use gelatin concentrations and reaction temperatures that disfavored triple-helix formation during coating and binding assays. The hexahistidine-tagged ColG variants bound to the immobilized soluble collagen or gelatin were detected using a rabbit polyclonal 6x His-tag antibody conjugated to HRP (Abcam, Austria). More details can be found in *SI Appendix*.

### Binding Assay to Fibrillar Collagen.

0.2 µM fluorescently labeled ColG-variants were incubated at 25 °C for 30 min with prewetted insoluble type I collagen from bovine Achilles tendon (Sigma, Germany) (0, 2, 4, 6, and 8 mg or 6 mg) in 50 mM HEPES pH 7.5, 100 mM NaCl, 10 mM CaCl_2_, 0.1% Tween-20, 1% fraction V of bovine serum albumin, 3 mM NaN_3_, ±10 mM ß-mercaptoethanol. The reactions were centrifuged at 13,000×*g* for 5 min at RT and the collagen-binding ability was determined by monitoring the free fluorescence intensity in the supernatant. More details can be found in *SI Appendix*.

### Binding Assay to V-(GXY)_26_ Monitored via Microscale Thermophoresis.

The detailed methods can be found in *SI Appendix*. MST experiments were performed with second-generation Monolith NT Protein Labeling Kit RED—NHS (NanoTemper Technologies, Germany) on a NanoTemper Monolith NT.115 instrument (NanoTemper Technologies, Germany). The assay buffer was 50 mM HEPES pH 7.5, 150 mM NaCl, 10 mM CaCl_2_, 10 µM ZnCl_2_, 0.05% Tween-20, 3 mM NaN_3_, ±1 mM ß-mercaptoethanol.

## Supplementary Material

Appendix 01 (PDF)

## Data Availability

All study data are included in the article and/or *SI Appendix*.
