# Supplementary material for: A conserved strategy to attack collagen: The activator domain in bacterial collagenases unwinds triple-helical collagen
Source: Proc Natl Acad Sci U S A. 2024 Apr 9;121(16):e2321002121. doi: 10.1073/pnas.2321002121 (PMC11032491; doi:10.1073/pnas.2321002121)
Supplement: Supplementary file 1 — Appendix 01 (PDF) [file pnas.2321002121.sapp.pdf]

PNAS

SI Appendix
A conserved strategy to degrade collagen: The activator domain in bacterial collagenases unwinds triple-helical collagen.

Jamil Serwanja [a,b], Alexander C. Wieland [a,b], Astrid Haubenhofer [a,b], Hans Brandstetter [a,b], Esther Schönauer [a,b]

[a] Department of Biosciences and Medical Biology, Paris-Lodron University of Salzburg, A-5020 Salzburg, Austria
[b] Center of Tumor Biology and Immunology (CTBI), Paris-Lodron University of Salzburg, A-5020 Salzburg, Austria

Corresponding author: Esther Schönauer
Email: esther.schoenauer@plus.ac.at

This PDF file includes:

SI Materials and Methods
Figures S1 to S16
Tables S1-S2
SI References

SI Materials and Methods

Disulfide Formation and Thiol Quantification Assay. For the crosslinkage, purified collagenases (0.1 mg/ml final concentration) were suspended in 50 mM Tris-HCl pH 8.5, 1 mM β-mercaptoethanol, 300 mM NaCl, 5% glycerol, 1 mM $CaCl_2$ and 3 mM NaN_3. The oxidation reactions were kept at 4 °C for 10 days followed by centrifugation (16,500 g for 20 min). The non-oxidized molecules were removed by Activated thiol sepharose™ 4B chromatography (Sigma Aldrich) performed according to the manufacturer's recommendations. In short, the clarified samples were loaded onto a self-packed activated thiol sepharose 4B column equilibrated in 50 mM Tris-HCl pH 8.5, 300 mM NaCl, 5% glycerol, 1 mM $CaCl_2$ and 3 mM NaN_3 and incubated overnight at 4 °C. The crosslinked variants were collected in the flowthrough fraction. The crosslinked monomers were separated from misoxidized aggregates by size exclusion chromatography using a Superdex 200 10/300 GL (Cytiva, Germany) and 10 mM HEPES pH 7.5, 100 mM NaCl, 5% glycerol and 3 mM NaN_3 as buffer. The extent of disulfide-bridge formation in the samples CL1-CL4 was examined using the thiol-specific fluorochrome 7-diethylamino-3-(4-maleinimidophenyl)-4-methyl coumarin (CPM) and ColG-CU WT (contains 2 cysteines) as positive control. CPM (Sigma-Aldrich, Germany) was dissolved at 4 mg/ml in DMSO and stored at -80 °C. Prior to use, the stock was diluted in the reaction buffer (10 mM HEPES pH 7.5 and 100 mM NaCl supplemented with 20% DMSO). The assay was performed in a total volume of 120 µL. The protein samples were diluted in the reaction buffer to 1.0 µM. 10 µL diluted dye and 16 µL DMSO were added to 94 µL protein solution. After 3 min of incubation at 60 °C for protein denaturation, the fluorescence was measured in an Infinite M200 plate reader (Tecan, Austria) at an excitation and emission wavelength of 387 nm and 463 nm, respectively.

Circular Dichroism Spectroscopy. Far UV circular dichroism (CD) spectra in the wavelength range from 195 to 260 nm were recorded using a Chirascan Plus CD Spectrophotometer (Applied Photophysics, Leatherhead, UK), equipped with a Peltier temperature-controlled cuvette holder at 25 °C in a 0.5-mm path length quartz cuvette. The instrument was flushed with nitrogen, the spectral bandwidth was set to 1 nm and the scan time per point 1 s. The samples (2.25 µM type I collagen, 5.0 µM V-$(GXY)_{26}$, 5.0 µM V, 2.25 µM and 10.0 µM ColG-CU G494V, 30.0 µM ColG-CBD2, 2.25 µM α-chymotrypsin, 10 µM ColG-CU G494V mutants, 10.0 µM CF, and 10.0 µM CL1-CL4) were measured in 15 mM Tris-SO_4 pH 7.5, 100 mM NaF, 1 mM $CaCl_2$ and ± 1 mM ß-mercaptoethanol. Melting profiles were measured at similar concentrations at a wavelength of 222 nm from 10 °C to 70 °C or 20 °C to 50 °C (setting time: 120 s, scan time per point: 1 s, step: 0.3 °C) and from 25 °C to 35 °C (setting time: 240 s, scan time per point: 10 s). To verify the proper folding of the samples, CD spectra from 195 to 260 nm were recorded at the start temperature prior to initiating the temperature ramp. In case of the melting temperatures of V-$(GXY)_{26}$ in absence or presence of ColG-CU G494V, ColG-AD and ColG-CBD2, the statistical significance was determined by one-way ANOVA followed by Dunnett's test for multiple comparisons (***P < 0.001). When comparing the melting temperatures of the V-$(GXY)_{26}$ in the presence of the different crosslinked variants, the statistical significance was determined by one-way ANOVA followed by Holm-Šídák's multiple comparisons test, comparing the reduced variant to its non-reduced control.

Peptidolytic Assay. The peptide-degradation assay was performed as described previously (40). In short, all ColG-CU variants were tested at a final concentration of 16 nM and co-incubated with 2 µM of the quenched-fluorescent peptidic substrate Mca-Ala-Gly-Pro-Pro-Gly-Pro-Dpa-Gly-Arg-NH2 (FS1-1) (Mca = (7-Methoxycoumarin-4-yl)acetyl; Dpa = N-3-(2,4-dinitrophenyl)-L-2,3-diaminopropionyl). The final reaction buffer contained 250 mM HEPES pH 7.5, 400 mM NaCl, 10 mM $CaCl_2$, 10 µM $ZnCl_2$, 2% DMSO and 3 mM NaN_3. Reactions were performed in the presence and absence of 0.5 mM TCEP. Cleavage of the substrate was monitored for 2 min at 25 °C (excitation: 328 nm, emission: 392 nm) in an Infinite M200 plate reader (Tecan, Austria) and the initial velocity (v_0) was determined from the progress curves (<10% substrate conversion).

Steady state measurements were used to determine K_M for FS1-1. The final concentration of ColQ1-CU WT and its variants was 1 nM and the concentration of the substrate was screened

from 0 – 175 µM. The initial velocity was determined from the progress curves (<10% substrate conversion) using linear regression and inner filter effect-correction (1). The ε_{ex328} and ε_{em392} were estimated in the buffer used for the kinetic assay to be 21794 M^{-1} cm^{-1} and 9561 M^{-1} cm^{-1}. K_M was calculated by non-linear regression from the resulting Michaelis − Menten plot using GraphPad Prism 9.1.2 (Graph Pad Software, San Diego, CA, USA).

Degradation of Fibrillar Collagen Monitored via Fluorescamine-Citrate Assay. Two milligrams of fibrillar collagen from bovine Achilles tendon (Merck, Germany) were added to a Nanosep microcentrifugal device (0.2 µm pore size) with a low-binding Bio-Inert membrane (Pall, Germany). 500 µL reaction buffer (250 mM HEPES pH 7.5, 400 mM NaCl, 10 mM $CaCl_2$, and 10 µM $ZnCl_2$, ± 10 mM ß-mercaptoethanol) were added for 15 min at room temperature to swell the fibers and then removed via centrifugation (13,000 g for 2 min). Then 200 µL 1.0 µM ColG-CU variants were added and incubated for 2 h at room temperature. The filtrate was collected and supplemented with 38 mM EDTA to stop the reaction. The amount of hydrolysis was quantified in comparison to the control reaction with ColG-CU WT exploiting the N-terminal-specific adduction of fluorescamine to peptides proteins at mildly acidic pH (47). In short, 5 µL of the stopped reaction were diluted 1:10 with reaction buffer and then mixed 1:1 with 1 M citrate pH 5.6. 100 µL of this mixture were added to 10 µL 2.5 mg/ml fluorescamine in acetone and incubated for 5 min at room temperature, before the fluorescence was measured at 25 °C (excitation: 390 nm, emission: 475 nm) in an Infinite M200 plate reader (Tecan, Austria). All experiments were performed at least in triplicates.

Indirect ELISA. Since gelatin can partially refold into triple-helical structures upon cooling (48), particular care was taken to use gelatin concentrations and reaction temperatures that disfavored triple-helix formation during coating and binding assays. For the coating, 1 mg/ml type I atelocollagen stock solutions were prepared in 0.1 M HCl and diluted to 5 µg/ml in 20 mM phosphate buffer pH 7.4, 150 mM NaCl. Gelatin solutions were prepared by heating type I collagen stock for 5 min at 95 °C. 96-well high-binding microplates (Greiner Bio-One, Germany) were incubated with 100 µL coating solution per well overnight at 4 °C for soluble collagen plates, and at 37 °C for gelatin plates. After incubation, the plates were washed four times with PBST. Coated wells were blocked with 1x PBS supplemented with 10% skim-milk for 90 min and then washed four times with PBST. Soluble collagen plates were stabilized with PBST supplemented with 1% BSA fraction V and 5% sucrose. Plates were dried and stored at 4 °C. For the binding assay, the hexahistidine-tagged ColG variants were prepared in PBST supplemented with 1% BSA fraction V. For K_D determination, the samples were serially diluted 1:3. 75 µL sample per well were incubated for 140 min with collagen at room temperature, while gelatin plates were incubated at 37 °C for the same time period. The plates were washed four times with PBST and were then incubated with 1:15,000 rabbit polyclonal 6x His-tag antibody conjugated to HRP (Abcam, Austria) for 1 h at room temperature, followed by four washes with PBST and a final wash with 1x PBS. As substrate 75 µL 3,3′,5,5′-tetramethylbenzidine were added per well. The peroxidase activity was followed by measuring the absorption at 650 nm every 15 s for 225 s in an Infinite M200 plate reader (Tecan, Austria) at 25 °C. The initial velocity was determined by regression analysis. For K_d determination, the data was fitted to the Hill equation using GraphPad Prism 9 (Graph Pad Software, USA). The apparent dissociation constant K_d is given as mean values of three independent experiments ± standard deviation. Statistical significance was determined by one-way ANOVA followed by Dunnett’s test for multiple comparisons (* $P < 0.05$, *** $P < 0.001$, **** $P < 0.0001$).

Binding Assay to Fibrillar Collagen. ColG-variants were labeled using the Monolith NT™ Protein Labeling Kit RED-NHS 2nd Generation Amine reactive (NanoTemper, Germany) and the yield of labelled protein and the degree of labelling were determined according to the manufacturer’s manual. Insoluble type I collagen from bovine Achilles tendon (Sigma, Germany) (0, 2, 4, 6, and 8 mg or 6 mg) was prewetted with 225 µL reaction buffer (50 mM Hepes pH 7.5, 100 mM NaCl, 10 mM $CaCl_2$, 0.1% Tween-20, 1% fraction V of bovine serum albumin, 3 mM NaN_3, ± 10 mM ß-mercaptoethanol) for 15 min at RT, before 100 µL labelled ColG-variants solubilized in reaction buffer were added. The final concentration of labelled protein was 0.2 µM,

except for ColG-PKD, which was added at 5 µM final concentration to ensure a proper signal-to-noise ratio. The reactions were incubated at 25 °C for 30 min with stirring in the dark and then centrifuged at 13,000 g for 5 min at RT. Labelled ColG variants in reaction buffer without any substrate were used as controls. The fibrillar collagen-binding ability of labelled proteins was determined monitoring the free fluorescence intensity in the supernatant after incubation using a Tecan M200 Infinite plate reader (Tecan, Austria) (647 nm excitation/680 nm emission).

Binding Assay to V-(GXY)$_{26}$ Monitored via Microscale Thermophoresis. Microscale thermophoresis experiments were performed on a NanoTemper Monolith NT.115 instrument (NanoTemper Technologies). ColG variants were labeled using the Monolith NT™ Protein Labeling Kit RED-NHS 2nd Generation Amine reactive (NanoTemper Technologies, Germany). After labeling, the ColG variants were eluted into 250 mM Hepes pH 7.5, 150 mM NaCl, 10 mM $CaCl_2$, 10% glycerol, 3 mM NaN_3 and stored at -80 °C. The yield of labelled protein and the degree of labelling were determined according to the manufacturer's manual. For the assay, the labelled proteins and the ligand were diluted into 50 mM Hepes pH 7.5, 150 mM NaCl, 10 mM $CaCl_2$, 10 µM $ZnCl_2$, 0.05% Tween-20, 3 mM NaN_3, ± 1 mM ß-mercaptoethanol. The ColG variants were used at a concentration of final concentration of 25 nM (except for 9xAla-Linker where 40 nM were used because of the low degree of fluorescent labelling), while the ligand was titrated in a 1:2 dilution series. After that ColG variants and ligand were mixed 1:2 and the samples were centrifuged for 10 min at 17,000 g at RT. The solutions were immediately transferred into Monolith NT.115 standard capillaries and measured using 60% excitation power at 22 °C. The binding of the ligand caused a reduction in the initial fluorescence signal, confirmed via specificity test performed according to the manufacturer's guidelines. The experiments were performed in triplicates. The change in the initial fluorescence signal was used to calculate the apparent binding constant K_d using non-linear regression analysis in GraphPad Prism 9 (Graph Pad Software, USA).

Rational Design and Production of Crosslinked ColG-CU Variants. To ensure efficient disulfide-bridge formation, we were looking for non-conserved residues on the inner-facing surfaces of the AD and PD. We generated a model of (semi)-closed conformations of ColG-CU based on PDB entry 2y50 using PYMOL software (2) and identified two residue pairs Y280/Q512 (mutant CL2), and E294/T483 (mutant CL3) for the introduction of cysteines in the upper half of ColG-CU, located at varying distances from the linker region (**Fig. 5A-B**). For mutant CL4, cysteines were introduced in a loop of ColG-PD and just before the N-terminus, in order to crosslink the ColG-CU at the tips of the AD and PD domains, locking the CU in a closed conformation. The mutants CL2-CL4 were generated on the basis of a cysteine-free ColG-CU (C218S/C262S) (mutant CF).

All ColG-CU constructs yielded over 20 mg of homogenous monodisperse protein after purification and oxidation from two liters of *E. coli* cell culture. They migrated with an apparent molecular mass of 79 kDa on a denaturing non-reducing SDS-PAGE gel and were estimated to be approximately 95% pure (**Fig. S15A**). SDS-PAGE analysis revealed that there were negligible amounts of oligomeric forms of the crosslinked mutants, indicating the robustness of the crosslinking approach, and we confirmed that the oxidation process did not negatively affect the collagenolytic activity *per se* (**Fig. S11C**).

The presence of the disulfide linkage was confirmed by a thiol quantitation assay (**Fig. S15B**). All crosslinked ColG-CU variants were tested at 1 µM concentration. ColG-CU WT which harbors two buried cysteines in the AD was used as positive control and we could confirm the presence of the disulfide bonds in CL2 to CL4. Non-reducing CD spectroscopy analysis showed that all mutants had a secondary structure similar to ColG-CU WT, suggesting that the formation of the disulfide bond did not compromise the overall fold (**Fig. S15C**). Finally, we compared the activity of the mutants towards a small quenched-fluorescence peptide substrate to the activity of ColG-CU WT to confirm the proper folding of the PD in the crosslinked variants (**Fig. S15D**). The removal of the two native cysteines in the AD in CF did not compromise its peptidolytic activity and collagenolytic activity (**Fig. S12**). In CL3 and CL4, the additional introduction of the cysteines for crosslinking also did not inhibit peptide hydrolysis in the reduced state (86 ± 19%, 105 ± 16%, respectively) and in the oxidized state, when the crosslink was established (78 ± 8%, and 113 ±

14%, respectively). However, mutant CL2 showed a notably reduced substrate turnover in both states (47 ± 10% and 27 ± 4% residual activity under non-reducing and reducing conditions, respectively).

Model Generation for the Complex of ColG-FL with a Mini Collagen Triple-Helix Using Protein–Protein Docking. A full-length model of ColG was generated using Alphafold2 (3). MMseqs2 and HHsearch with the PDB100 option were used to generate templates, thereby integrating the available structural information of the single ColG domains into the Alphafold predictions. The resulting five relaxed models were manually curated. The Alphafold model which positioned the CBD domains correctly behind the CU was used for further docking. Alphafold2 was also used to generate a mini collagen triple helix based on the sequence $(GPP)_{60}$. Protein-protein docking of ColG-FL to the $(GPP)_{60}$ triple helix was performed using the HADDOCK 2.4 server (4, 5), as this docking method allows for the definition of known binding sites as input for the docking procedure. The active site/interface residues of the AD (F148, E191, Y198, N251), CBD1 (Y950, H958, F983, Y985, H987) and CBD2 (L1034, S1038, Y1080, L1102, Y1104, Y1106) were given as input. In addition, the linker regions between the AD and PD (389-397) and between CBD1 and CBD 2 (999-1004) were defined as fully flexible. The resulting complex models were manually curated.

Figures

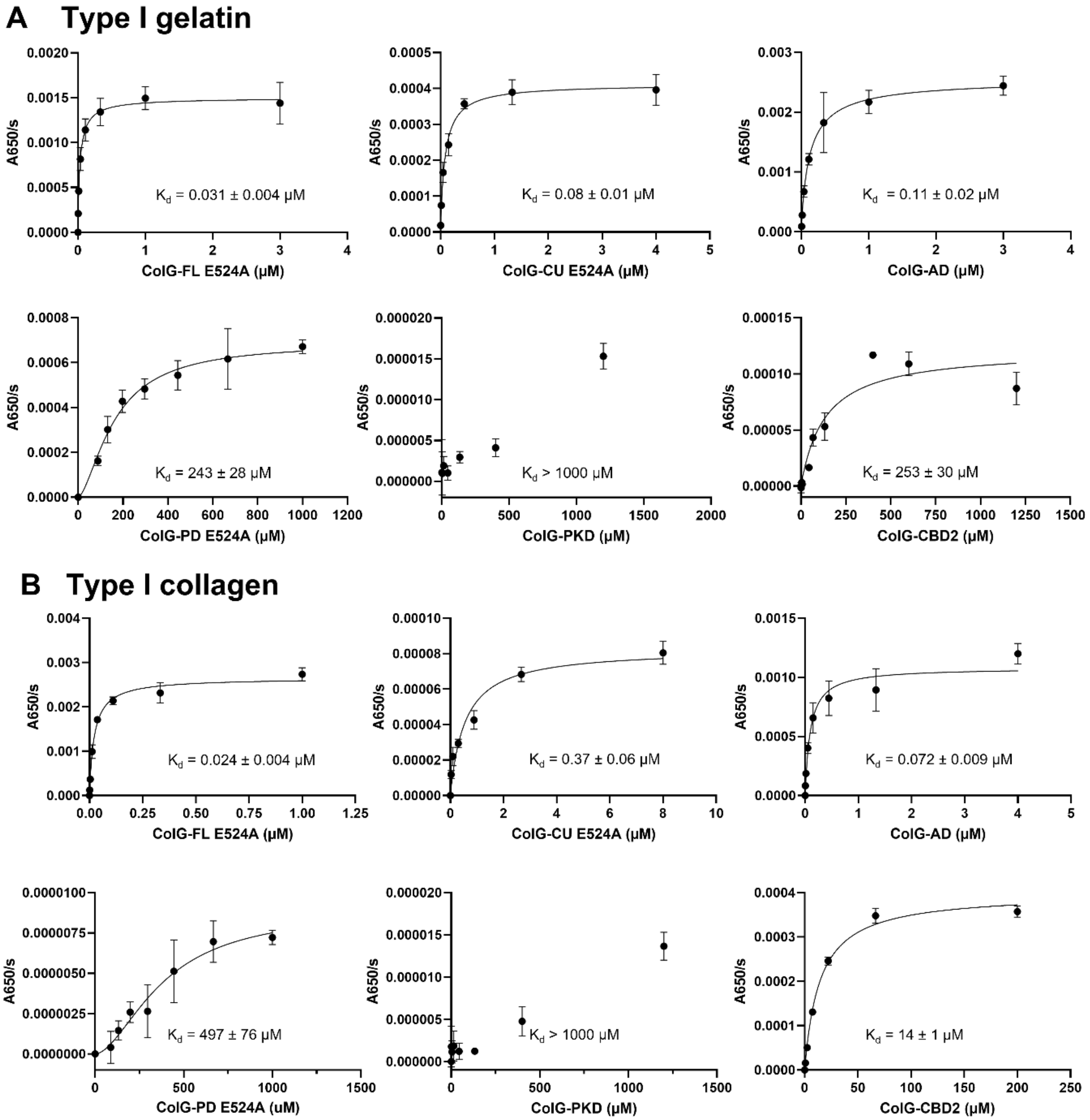

Fig. S1: ELISA binding assays. A, Binding curves of ColG variants measured by indirect ELISA on plates coated with gelatin. Incubation was performed at 37 °C to prevent partial refolding of collagen. **B**, Binding curves of ColG variants to soluble collagen. Incubation was performed at 25 °C to prevent collagen unfolding.

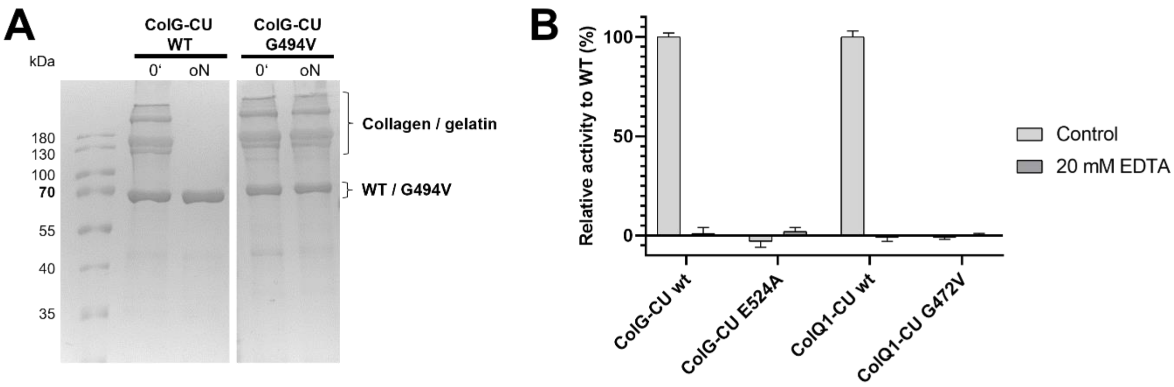

Fig. S2: ColG-CU G494V, ColG-CU E524A and ColQ1-CU G472V are inactive mutants. A, 3.33 µM soluble collagen was digested at 25 °C by 4.54 µM ColG-CU variants overnight. Samples were taken at indicated time points and the reaction was stopped by addition of 38 mM EDTA. **B**, ColG-CU and ColQ1-CU variants were incubated with 2 µM FS1-1, a fluorescent-quenched peptide custom-tailored for collagenases, and cleavage was monitored via fluorescence at 328/392 nm at RT.

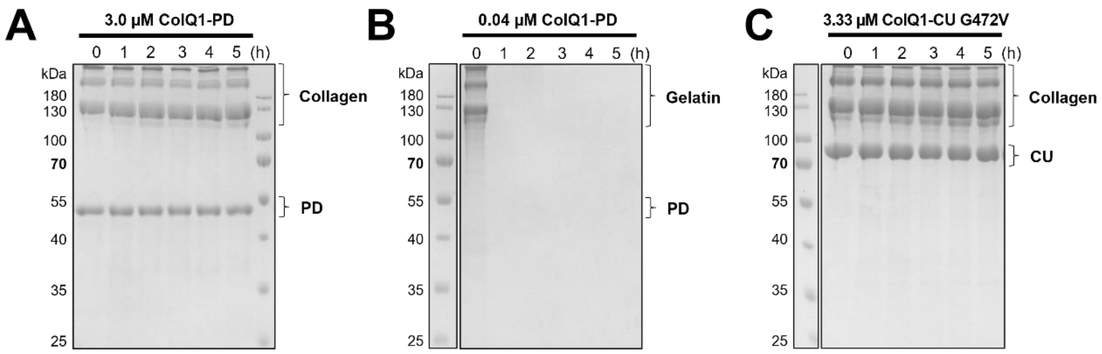

Fig. S3: ColQ1-PD is an efficient gelatinase, but not collagenase. ColQ1-PD was incubated with 3.33 µM soluble collagen (**A**) or gelatin (**B**) at 25 °C or 37 °C, respectively. **C**, ColQ1-CU G472V was incubated with 3.33 µM soluble collagen at 25 °C. Samples were taken at indicated time points and the reaction was stopped by addition of 38 mM EDTA.

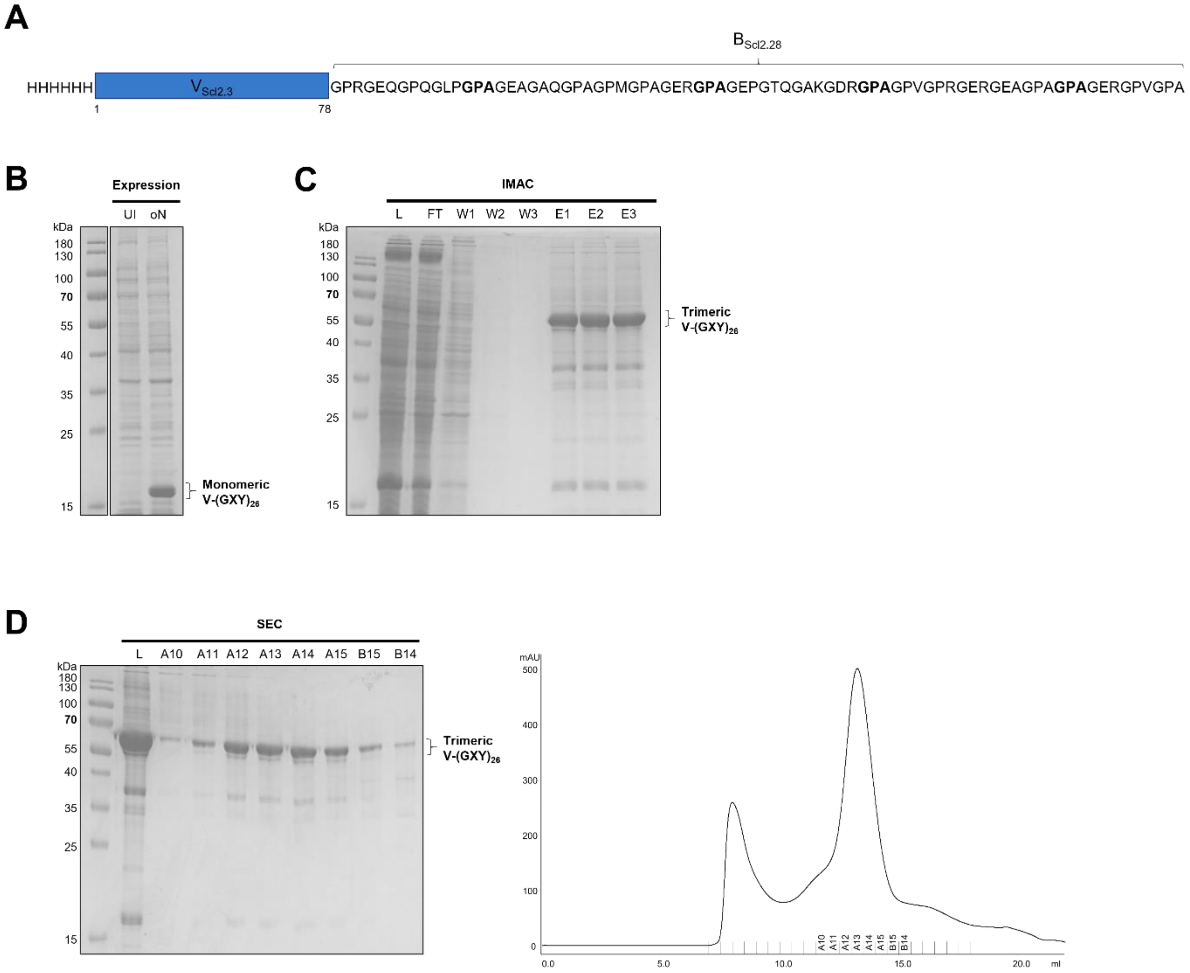

Fig. S4: Organization and production of V-(GXY)$_{26}$. A, Scheme of V-(GXY)$_{26}$. Mutations in segment B are indicated in bold. **B**, SDS-PAGE analysis of overnight expression culture at 25 °C in E. coli Nico21 (DE3). Uninduced cell lysate (UI) and cell lysate after overnight expression (oN). **C**, Purification of His-tagged V-(GXY)$_{26}$ via immobilized metal-affinity chromatography (IMAC) using Nickel-Sepharose. **D**, Size-exclusion chromatography (SEC) of His-tagged V-(GXY)$_{26}$ using a Superdex 200 10/300 GL column monitored by SDS-PAGE. *L*, load; *FT*, flow-through; *W1*-*W3*, wash fractions 1-3; *E1*-*E3*, elution fractions 1-3.

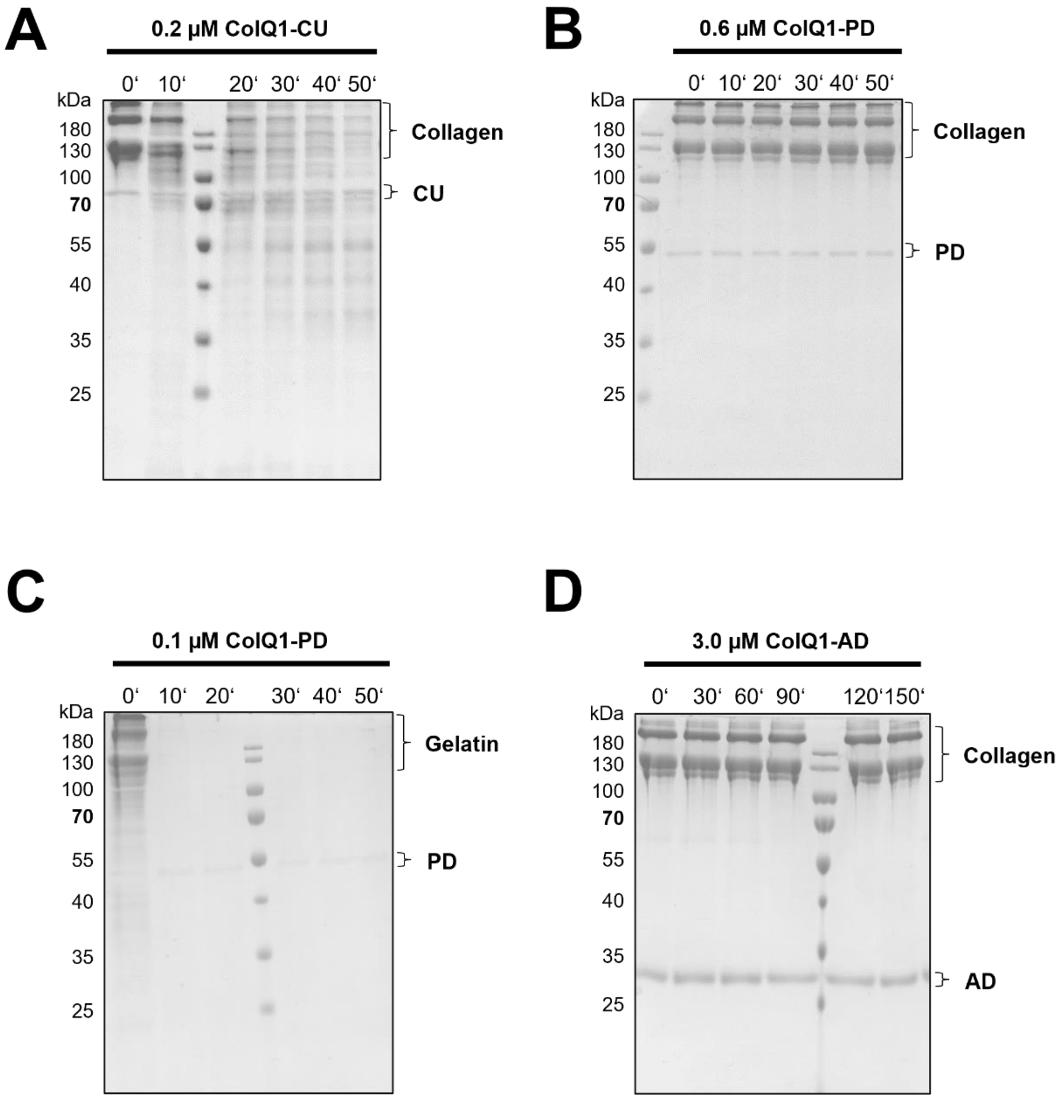

Fig. S5: ColQ1-CU is an efficient collagenase, whereas ColQ1-PD is an efficient gelatinase, but not collagenase. A, Degradation of 3.33 µM soluble collagen by 0.2 µM ColQ1-CU at 25 °C within 50 min. **B**, Incubation of 3.33 µM soluble collagen with 0.6 µM ColQ1-PD at 25 °C did not result in any detectable collagen degradation within 150 min, while in **C** 3.33 µM gelatin (generated by heat denaturation of soluble collagen at 95 °C for 5 min) were completely turned over within the first 30 min by 0.1 µM ColQ1-PD at 37 °C. **D**, 3.33 µM soluble collagen were stable in the presence of 3.0 µM ColQ1-AD at 25 °C. Reactions were terminated by addition of 35.6 mM EDTA and subjected to SDS–PAGE under non-reducing conditions.

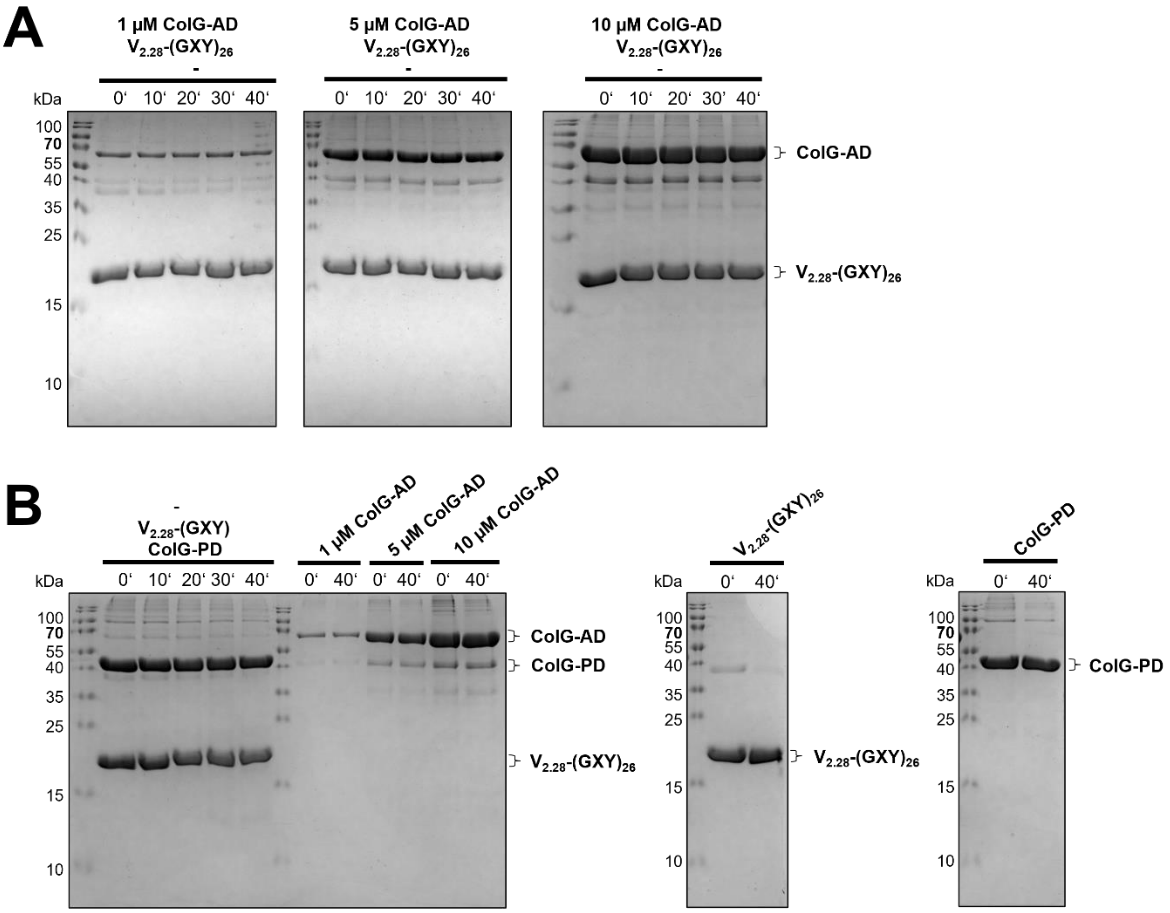

Fig. S6: The individual AD and PD cannot degrade triple-helical V-(GXY)$_{26}$. A, Co-incubation of 10 µM V$_{2.28}$-(GXY)$_{26}$ with 1, 5 or 10 µM ColG-AD-MBP. **B**, Co-incubation of 10 µM V$_{2.28}$-(GXY)$_{26}$ with 10 µM ColG-PD and single protein control samples. All reactions were performed at 25 °C. The reactions were stopped by the addition of SDS-loading buffer and analyzed by SDS-PAGE.

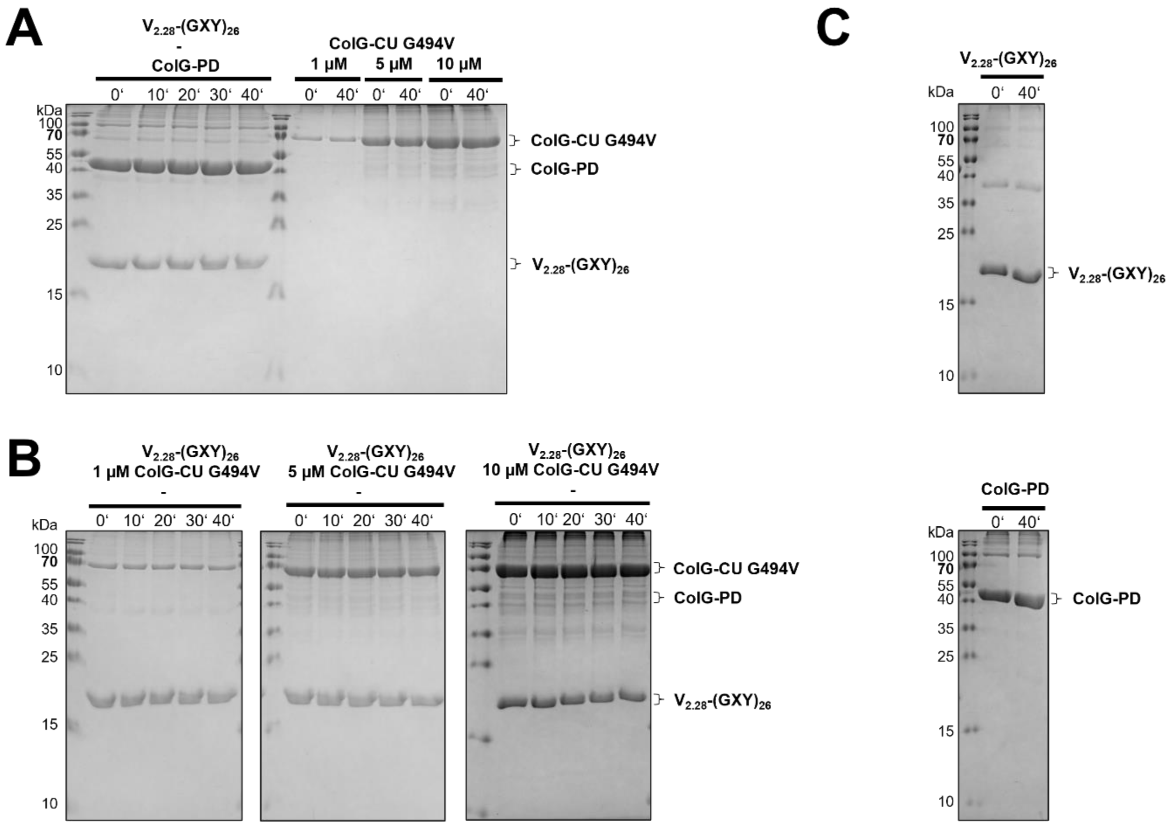

Fig. S7: Control reactions. A, Co-incubation of 10 µM $V_{2.28}$-$(GXY)_{26}$ with 10 µM ColG-PD. **B,** Co-incubation of 10 µM $V_{2.28}$-$(GXY)_{26}$ with 1, 5 or 10 µM ColG-CU G494V. **C,** Single protein control samples: 10 µM $V_{2.28}$-$(GXY)_{26}$ and 10 µM ColG-PD. All reactions were performed at 25 °C. The reactions were stopped by the addition of SDS-loading buffer and analyzed by SDS-PAGE.

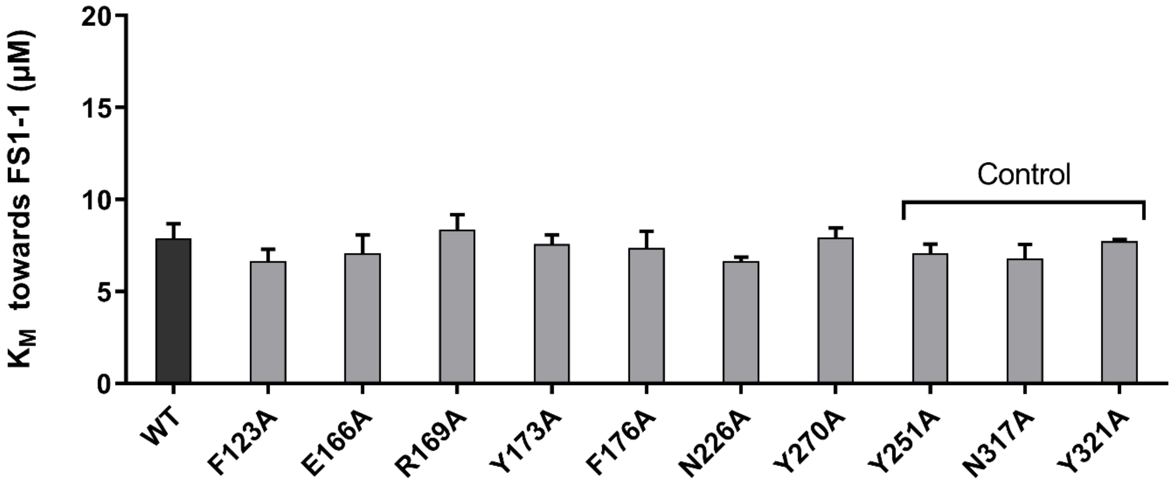

Fig. S8: Michaelis-Menten constants of ColQ1-CU WT and its single-point mutants towards peptide FS1-1. Steady state measurements were performed using ColQ1-CU WT and its single-point mutants at 1 nM concentration. The substrate concentration was varied from 0 – 175 µM. Initial velocities were determined using linear regression and the K_M was calculated by non-linear regression using GraphPad Prism 9.1.2 (Graph Pad Software, San Diego, CA, USA). Mutants Y251A, N317A and Y321A were used as controls.

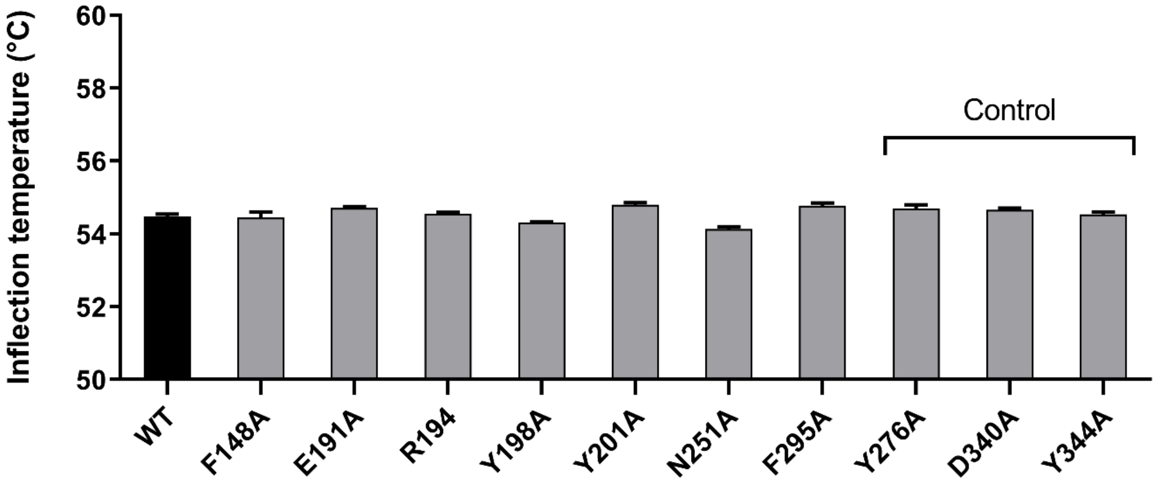

Fig. S9: Thermal stability of ColG-CU G494 WT and its variants. Thermal denaturation profiles were measured at 0.1 mg/ml in 15 mM Tris-SO4 pH 7.5, 100 mM NaF and 1 mM $CaCl_2$ using a Tycho NT.6 (Nanotemper, Germany) exploiting the intrinsic fluorescence of tryptophane and tyrosine residues detected at 350 nm and 330 nm. Mutants Y276A, D340A and Y344A were used as controls.

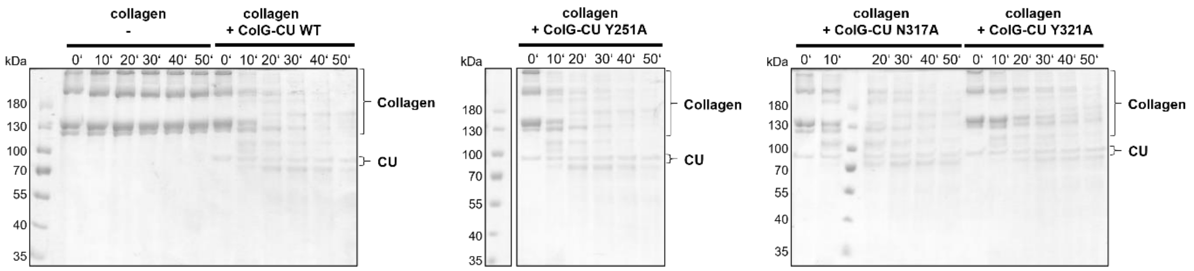

Fig. S10: Activity of ColQ1-CU WT control mutants towards soluble collagen. 3.33 µM soluble collagen were incubated with 0.2 µM ColQ1-CU variants at 25 °C. The reactions were stopped by the addition of 38 mM EDTA and SDS-loading buffer.

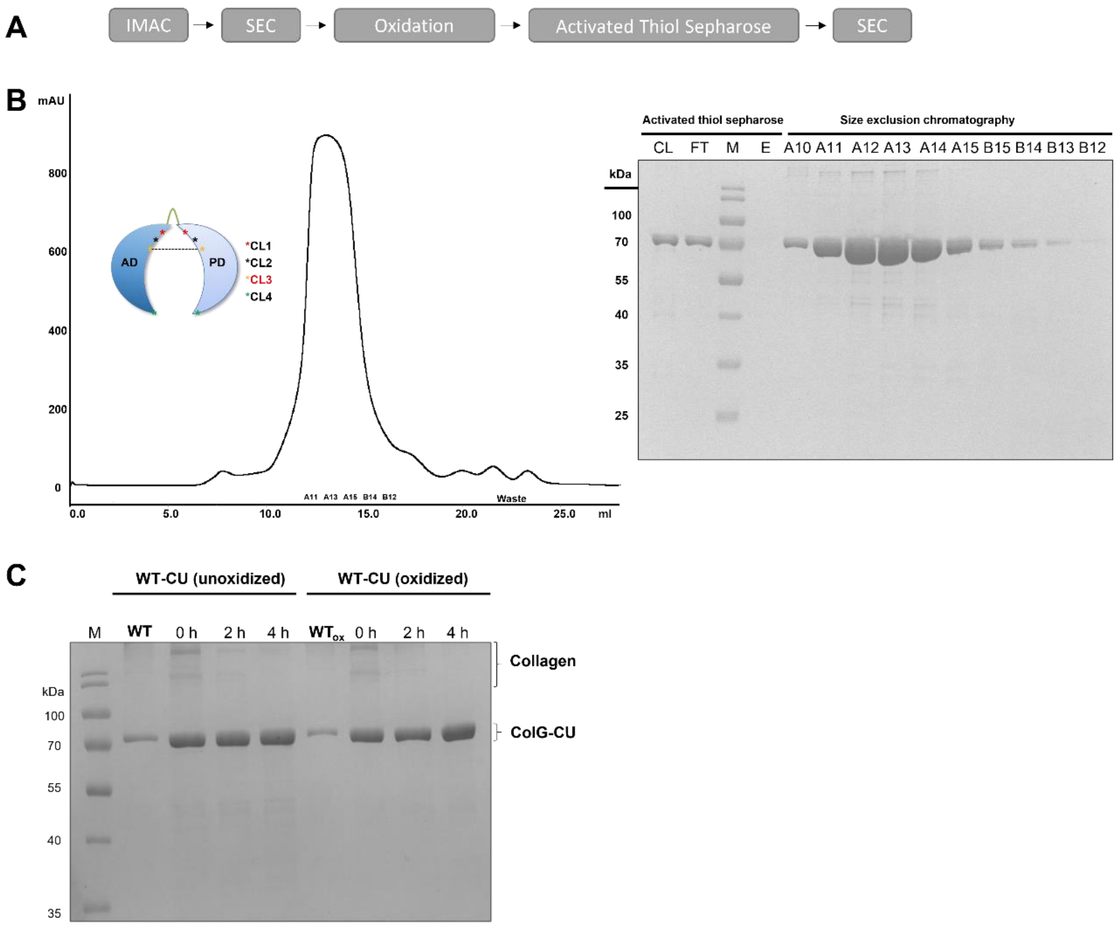

Fig. S11: Purification of crosslinked ColG-CU mutants. A, Scheme of production workflow. After metal-affinity purification (IMAC) and a first size-exclusion chromatography (SEC) under reducing conditions, the purified proteins were air-oxidized over 10 d at 4 °C. Non-oxidized species were removed via the Activated Thiol Sepharose resin and misoxidized aggregates were removed by SEC. **B**, Representative example of the final polishing SEC plus corresponding SDS-PAGE analysis including the results of the Activated Thiol Sepharose chromatography. **C**, Oxidation procedure does not affect collagen degradation by ColG-CU WT. ColG-CU WT was stored for 10 d at 4 °C under reducing conditions or air-oxidized over 10 days at 4 °C and then purified by SEC. The purified proteins were tested vs. 1 µM soluble collagen at 25 °C.

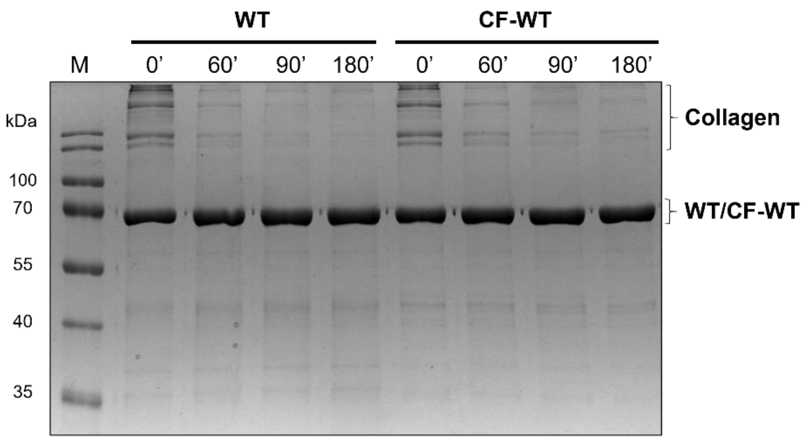

Fig. S12: CF-WT shows similar collagenolytic activity like ColG-CU WT. 3.33 µM soluble collagen was digested at 25 °C by 4.54 µM collagenase for up to 4 h. Samples were taken at indicated time points and the reaction was stopped by addition of 38 mM EDTA. The integrity of the collagen fold was verified by co-incubation with 0.83 µM α-chymotrypsin.

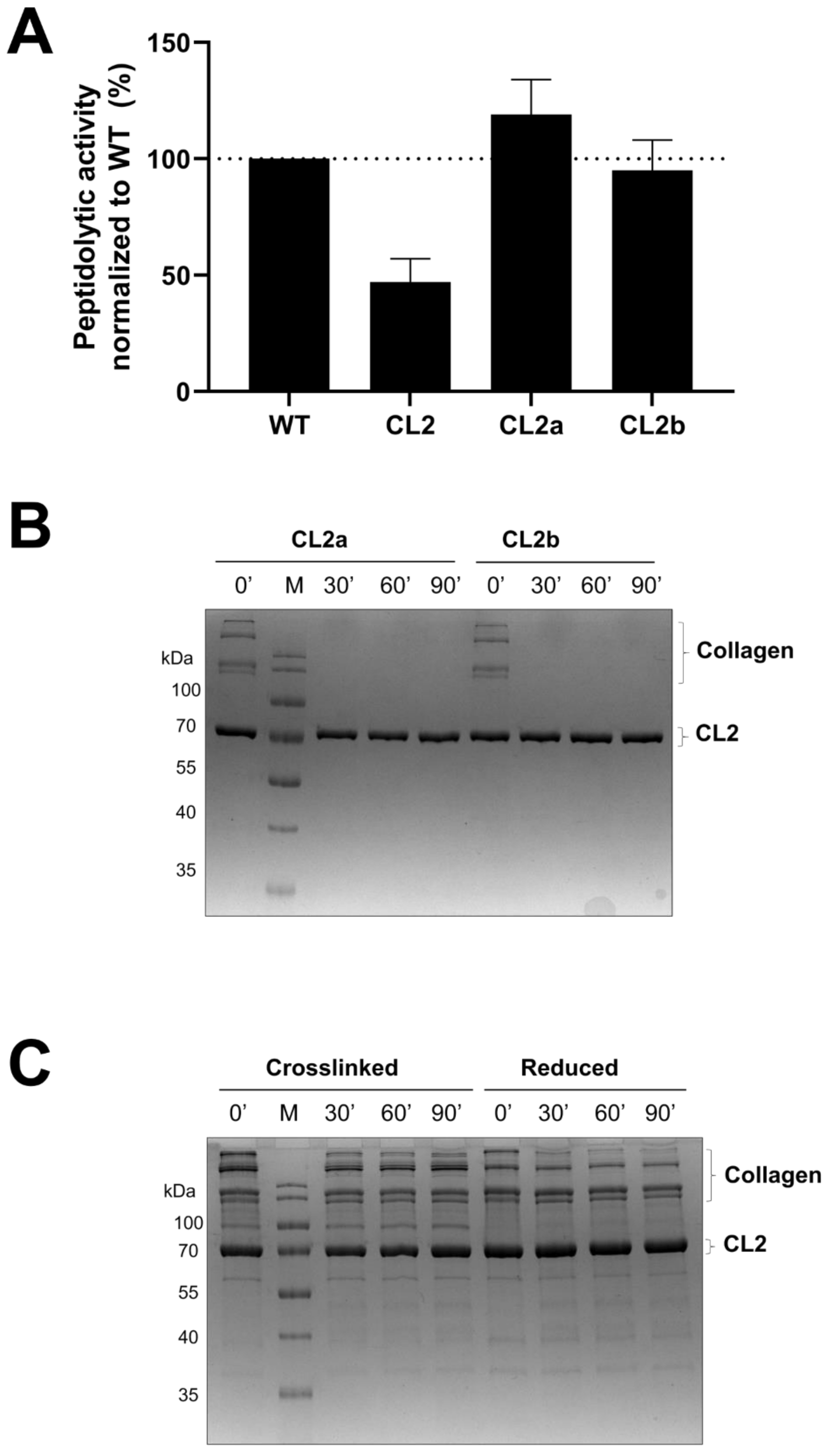

Fig. S13: Peptidolytic and collagenolytic activity of CL2 and its variants. A, Activities of 16 nM ColG-CU WT, CL2, CL2a and CL2b towards 2 µM quenched-fluorescence substrate. The specific activity of ColG-CU WT was taken as 100%. **B**, Activity of the single-point mutants CL2a and CL2b towards soluble collagen over 90 min at 25 °C under non-reducing conditions. **C**, Activity of CL2 towards soluble collagen over 90 min at 25 °C under non-reducing and reducing conditions, separated on a non-reducing 12% SDS-PAGE.

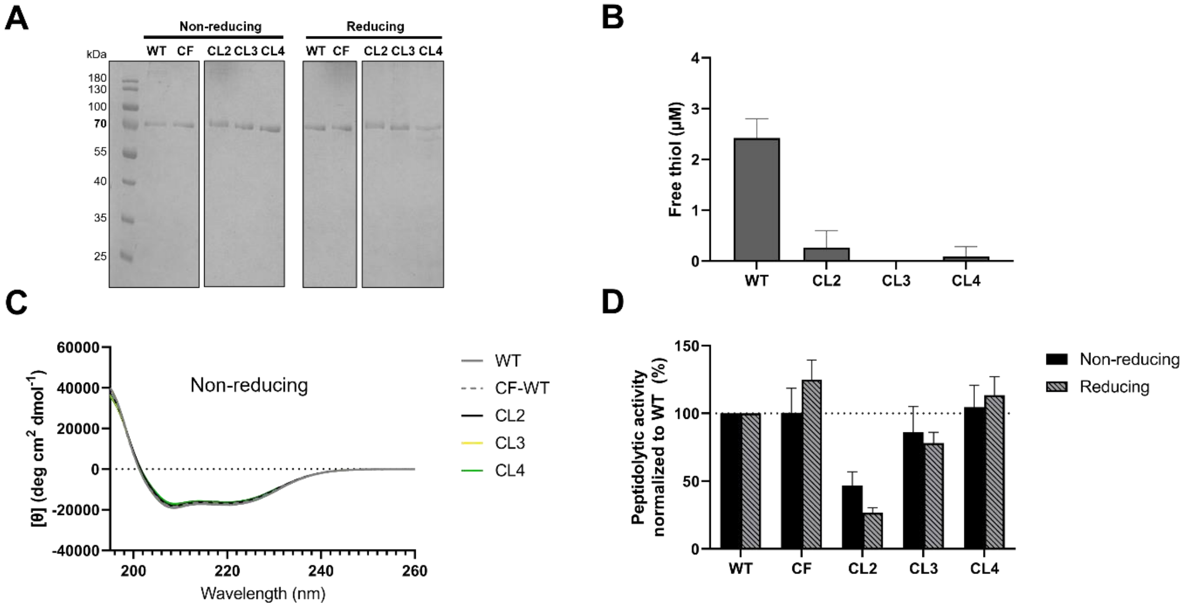

Fig. S14: Quality control of crosslinked variants CL2-CL4 of ColG-CU. A, SDS-PAGE analysis of ColG-CU WT, CF and the mutants CL2-CL4 under non-reducing and reducing conditions on a 12% polyacrylamide gel. **B**, The presence of free thiols was detected using the thiol-specific fluorochrome 7-diethylamino-3-(4-maleinimidophenyl)-4-methyl coumarin after thermal denaturation of the variants at 60 °C. **C**, CD spectra of ColG-CU WT and the mutants CL2-CL4 in the absence of reducing agent. The data shown are representative of triplicate experiments. **D**, Peptidolytic activity of ColG-CU WT compared to CF and the crosslinked mutants under reducing and non-reducing conditions. 16 nM ColG-CU variants were co-incubated with 2 µM quenched-fluorescent peptide FS1-1 in reaction buffer containing ± 0.5 mM TCEP.

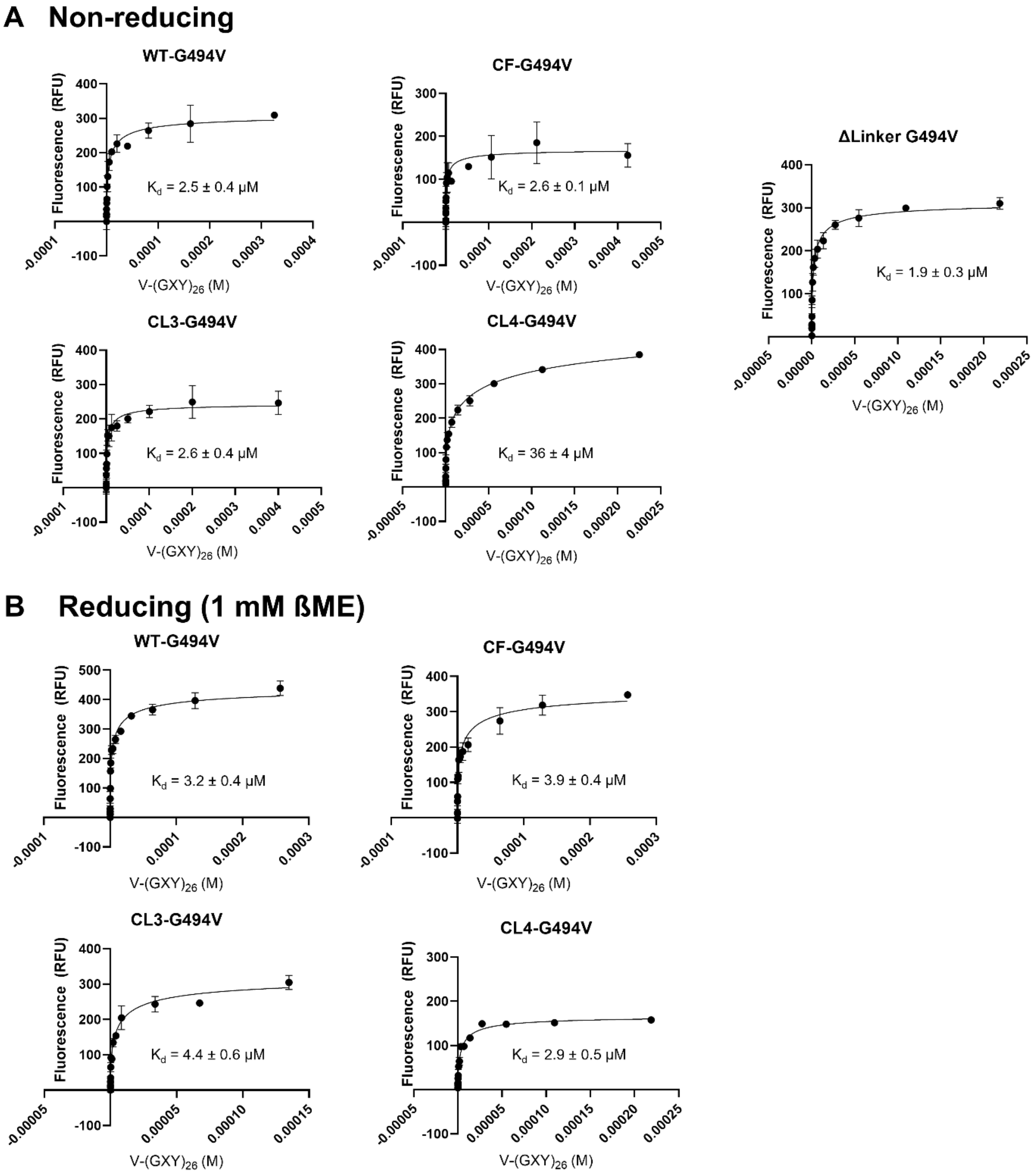

Fig. S15: MST binding curves. Binding curves of fluorescently labelled ColG variants towards triple-helical V-$(GXY)_{26}$ measured by microscale thermophoresis using the initial fluorescence signal. Experiments were performed at 22 °C in the absence (**A**) or presence (**B**) of 1 mM ß-mercaptoethanol.

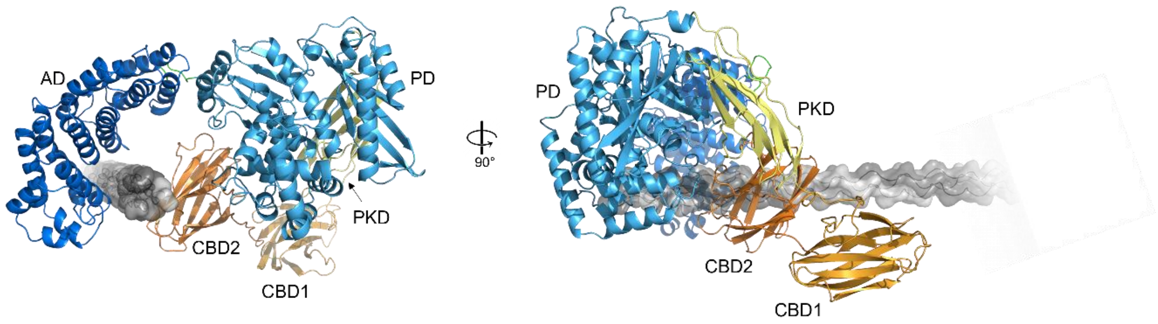

Fig. S16: Docking model of ColG-FL on collagen triple helix. Ribbon and surface representation of ColG-FL and a mini collagen triple helix composed of $(GPP)_{60}$. The structure models were generated with Alphafold2 and then docked with HADDOCK 2.4, an information-driven docking tool. Although binding residues for AD, CBD1 and CBD2 were defined, the docking only positioned the AD and CBD2 on the triple helix. The latter docking is in line with the findings that i) the linker between CBD1 and CBD2 does not allow for both domains to bind simultaneously to the same collagen molecule (6) and ii) that the CBD2 domain exhibits the higher affinity to soluble collagen than CBD1 (7). The structures of the individual CU, PKD and CBD2 of the docked ColG-FL structurally superimpose well with their respective crystal structures (RMSD = 1.405 / 0.747 / 0.588, PDB: 2y50 / 2y72 / 4HPK calculated using Pymol (2)). The same color code for ColG as in Fig. 1 is used.

Table S1: Dissociation constants (K_d) of ColG variants towards gelatin and soluble collagen determined by ELISA.

	**Gelatin** K_d (mean ± SD) (µM)	**Soluble collagen** K_d (mean ± SD) (µM)
**FL E524A**	0.031 ± 0.004	0.024 ± 0.002
**CU E524A**	0.08 ± 0.01	0.37 ± 0.06
**AD**	0.11 ± 0.02	0.072 ± 0.009
**PD E524A**	243 ± 28	497 ± 76
**PKD**	nd	nd
**CBD2**	253 ± 30	14 ± 1

The apparent dissociation constant K_d is given as mean value of three independent experiments ± standard deviation. nd, could not be determined.

Table S2: Dissociation constants (K_d) of ColQ1-CU G494V mutants towards gelatin and soluble collagen determined by ELISA.

	Gelatin<br>K_d (mean ± SD) (µM)	Soluble collagen<br>K_d (mean ± SD) (µM)
**WT**	1.6 ± 0.4	1.6 ± 0.2
**F123A**	1.6 ± 0.3	nbd
**E166A**	5.0 ± 0.6	nbd
**R169A**	2.8 ± 0.4	0.17 ± 0.04
**Y173A**	24 ± 6	nbd
**F176A**	1.2 ± 0.2	0.08 ± 0.01
**N226A**	> 150	> 150
**Y270A**	4.2 ± 0.5	0.19 ± 0.06
**Y251A**	2.5 ± 0.2	2.8 ± 0.6
**N317A**	2.3 ± 0.1	2.8 ± 0.5
**Y321A**	2.5 ± 0.1	4.0 ± 0.4

The apparent dissociation constant K_d is given as mean value of three independent experiments ± standard deviation. nbd, no binding detected.

SI References

1. Y. Liu et al., Use of a fluorescence plate reader for measuring kinetic parameters with inner filter effect correction. Anal. Biochem. 267, 331–335 (1999).

2. The PyMOL Molecular Graphics System, Version 2.0 Schrödinger, LLC (2010) New York (August 5, 2012).

3. M. Mirdita, *et al.*, ColabFold: making protein folding accessible to all. *Nat Methods* 19, 679–682 (2022).

4. R. V. Honorato, *et al.*, Structural Biology in the Clouds: The WeNMR-EOSC Ecosystem. *Frontiers in Molecular Biosciences* 8 (2021).

5. G. C. P. van Zundert, *et al.*, The HADDOCK2.2 Web Server: User-Friendly Integrative Modeling of Biomolecular Complexes. *Journal of Molecular Biology* 428, 720–725 (2016).

6. P. Caviness, *et al.*, Ca2+ Induced orientation of tandem collagen binding domains from clostridial collagenase ColG permits two opposing functions of collagen fibril formation and retardation. *FEBS J* 285, 3254–3269 (2018).

7. O. Matsushita, T. Koide, R. Kobayashi, K. Nagata, A. Okabe, Substrate Recognition by the Collagen-binding Domain of Clostridium histolyticum Class I Collagenase. *Journal of Biological Chemistry* 276, 8761–8770 (2001).
